# The upconversion quantum yield (UCQY): a review to standardize the measurement methodology, improve comparability, and define efficiency standards

**DOI:** 10.1080/14686996.2021.1967698

**Published:** 2021-12-17

**Authors:** Callum M. S. Jones, Anna Gakamsky, Jose Marques-Hueso

**Affiliations:** a Institute of Sensors, Signals and Systems, Heriot-Watt University, Edinburgh, UK; b Edinburgh Instruments Ltd., Livingston, UK

**Keywords:** Upconversion quantum yield, UCQY, photoluminescence quantum yield, PLQY, photoluminescence efficiency characterization, upconversion phosphors, upconversion nanoparticles, NaYF_4_, lanthanides, rare-earth dopants, 40 Opticalmagnetic and electronic device materials, 102 Porous/Nanoporous/Nanostructured materials, 107 Glass and ceramic materials, 204 Optics/optical applications, 206 Energy conversion/transport/storage/recovery, 505 Optical/molecular spectroscopy

## Abstract

Advancing the upconversion materials field relies on accurate and contrastable photoluminescence efficiency measurements, which are characterised by the absolute upconversion quantum yield (UCQY). However, the methodology for such measurements cannot be extrapolated directly from traditional photoluminescence quantum yield techniques, primarily due to issues that arise from the non-linear behaviour of the UC process. Subsequently, no UCQY standards exist, and significant variations in their reported magnitude can occur between laboratories. In this work, our aim is to provide a path for determining and reporting the most reliable UCQYs possible, by addressing all the effects and uncertainties that influence its value. Here the UCQY standard, at a given excitation power density, is defined under a range of stated experimental conditions, environmental conditions, material properties, and influential effects that have been estimated or corrected for. A broad range of UCQYs reported for various UC materials are scrutinized and categorized based on our assertion of the provided information associated with each value. This is crucial for improved comparability with other types of photoluminescent materials, and in addition, the next generation of UC materials can be built on top of these reliable standards.

## Graphical abstract

**Figure:**
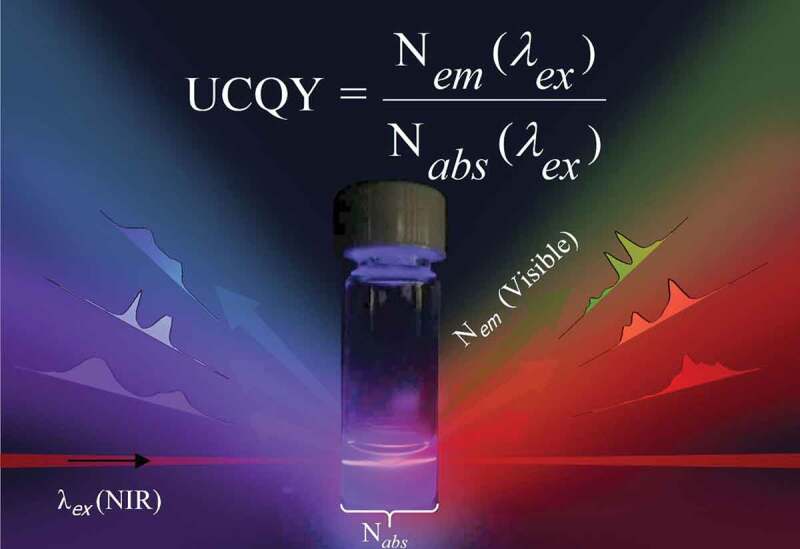

## 1. Introduction

### 1.1. Upconversion materials and the demand for reliable characterization

The upconversion (UC) process consists of the absorption of low energy photons and the subsequent emission of a relatively high energy photon. François Auzel pioneered the research into UC materials and developed an understanding of their unique photoluminescence [1]. This can occur through various mechanisms involving Rare-Earth trivalent ions (RE^3+^), which are commonly co-doped into an inorganic dielectric host material that possesses low phonon energies [2,3]. The most basic of mechanism involves a ground state absorption (GSA) process involving a low-energy photon, which promotes an electron to the first excited state. From here, excited state absorption (ESA) occurs, which pushes the electron to a second higher energy level through the absorption of another low-energy photon. Subsequently, when the electron relaxes to a lower energy level, a high-energy photon can be released through radiative decay. Alternatively, energy transfer upconversion (ETU) can occur between two neighboring RE^3+^ ions. ETU is more commonly exploited as it is regarded as the most efficient mechanism [4]. Here, one ion acts as a sensitizer by absorbing the excitation light, and through energy transfer mechanisms, it passes it to a nearby activator ion, which emits the UC light. Due to the strong resonance of the ^2^F_7/2_ → ^2^F_5/2_ transition, Yb^3+^ is frequently exploited as a sensitizer, as it can easily transfer this energy to ions such as Er^3+^ or Tm^3+^, which are common activators [5]. This transition is frequently targeted using 980 nm excitation due the strong absorption around this wavelength. For added context, the resulting transitions associated with the different ETU emissions are commonly categorized into the following wavelength regions [6]. For Er^3+^-doped samples: ^4^G_11/2_ → ^4^I_15/2_ (374–392 nm), ^2^H_9/2_ → ^4^I_15/2_ (396–428 nm), ^2^H_11/2_/^4^S_3/2_ → ^4^I1_5/2_ (511–581 nm), and ^4^F_9/2_ → ^4^I1_5/2_ (625–713 nm). Additionally, for Tm^3+^-doped materials: ^1^I_6_ → ^3^F_4_ (332–358 nm), ^1^D_2_ → ^3^H_6_ (358–373 nm), ^1^D_2_ → ^3^F_4_ (433–461 nm), ^1^G_4_ → ^3^H_6_ (461–502 nm), ^1^G_4_ → ^3^F_4_ (609–692 nm), and ^3^H_4_ → ^3^H_6_/^1^G_4_ → ^3^H_5_ (697–830 nm). It is important to note that due to the multi-photon nature of these mechanisms, UC materials exhibit a non-linear relationship between their emission power and the excitation power [7].^174–187^

**Figure 1.**
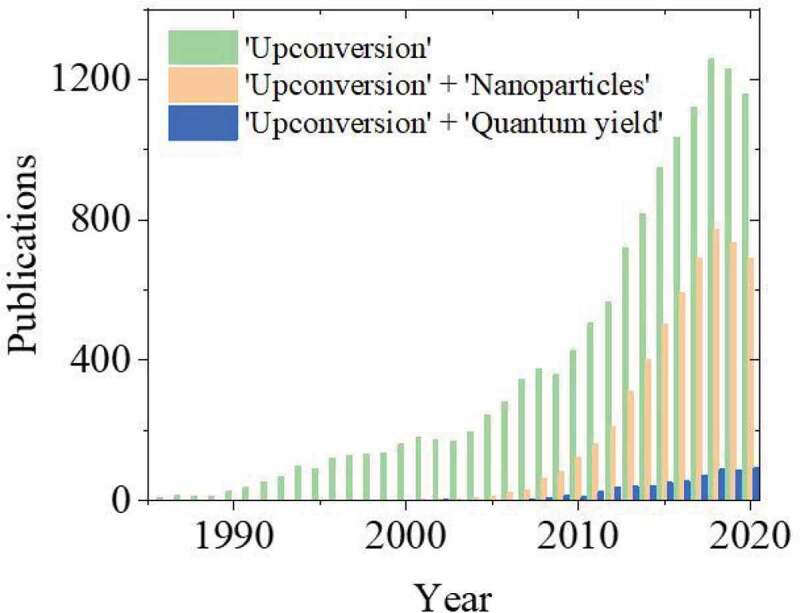
A histogram showing the number of publications per year that include the terms ‘Upconversion’, ‘Upconversion’ and ‘Nanoparticles’, as well as ‘Upconversion’ and ‘Quantum yield’

By carefully tailoring their compositions, UC materials can possess high photostability, long lifetimes, and high intensity narrow emissions in the visible/ultraviolet (UV) regions [8]. These properties give UC materials high application potential, which is further complemented by decreasing their size towards the nanoscale. However, despite their popularity, upconversion nanoparticles (UCNPs) have a considerably lower photoluminescence efficiency compared their bulk analogues, which is already limited due to the forbidden nature of the 4 f-4 f transitions that the UC mechanism relies on [9,10]. UCNPs also suffer due to their increased surface-to-volume ratio, poor crystallinity and surface defects, dopant spatial confinement, intrinsic photon mode, and quenching from their dispersion solvent [11,12]. There is a large movement in the field to produce UCNPs with higher photoluminescence efficiencies. Such avenues for achieving this include improving the host lattice selection [13], the combination and concentration of RE^3+^ dopants [14,15], improving their crystallinity [10], as well as introducing shell structures [16,17]. Such advancements have resulted in UCNPs being used for a wide range of applications such as anti-counterfeiting technology [18], bio-imaging and bio-sensing [2,19], drug delivery [20], theranostics [21], photodynamic therapy [22], nanothermometry [23], and photovoltaics [24]. Due to their rapidly growing popularity, UCNP research now encompasses over half of the UC literature that was published in 2020, as shown in Figure 1 [25]. Furthermore, plasmonic structures [26], photonic structures [27], and dye sensitizers [28], have also been exploited for photoluminescence efficiency enhancement in these materials, which is commonly characterized by the photoluminescence quantum yield (PLQY) measurement. However, as shown in Figure 1, the number of publications that include information on the PLQY of UC materials in relation to the total UC literature is relatively small. This review will address the various challenges associated with these measurements and compile a complete list of influential effects that interfere with their comparability. Previously reported values will be categorized based on their measurement parameters to give an indication of their reliability. Furthermore, avenues for obtaining UC efficiency standards with the highest comparability will be described.

### 1.2. Upconversion emission efficiency and the photoluminescence quantum yield

Auzel and Pecile were the first to characterise the photoluminescence efficiency of UC phosphor by dividing their emitted power by the incident excitation power used [29,30]. Similar methods have been used to report the optical efficiency [31,32], absolute efficiency [1,33], raw efficiency [34,35], and upconversion efficiency [36,37], among other related power ratio techniques [38–48]. As explained in the next paragraph, these types of measurements are less informative about the underlying energy transitions in the material in comparison to PLQY characterisations. Therefore, the PLQY will be the focus of this review.

First introduced by Vavilov when characterising organic dyes, the fluorescence quantum yield is defined as the number of photons emitted divided by the number of photons absorbed [49]. The term PLQY shares the same definition however, it is more generalised as it is defined as any photoluminescence from a species that results from its direct photoexcitation [50]. The PLQY measurement has evolved over the years with the improvement of technology, and it has been reviewed on various occasions due to its importance as a spectroscopic tool in the field of photoluminescent materials [51–54]. It’s regularly used to characterise materials such as organic dyes [55], fluorescent proteins [56], quantum dots [57,58], metal nanoclusters [59], conjugated polymers [60], metal-ligand complexes [61], carbon nanotubes [62], and UC materials [6]. In regards to the later, interchangeable PLQY nomenclature has been used such as the IQY [63], AQY [64], Φ [65], Φ_UC_ [15], Φ_UCFL_ [66], QY_UC_ [10], UC-PLQY [67], and upconversion quantum yield (UCQY) [68]. For clarity, this review will use the term UCQY. This will also help to differentiate between procedures and influential effects that are important for UCQY characterisations but not traditional PLQY measurements.

### 1.3. Determining the upconversion quantum yield (UCQY)

**Figure 2.**
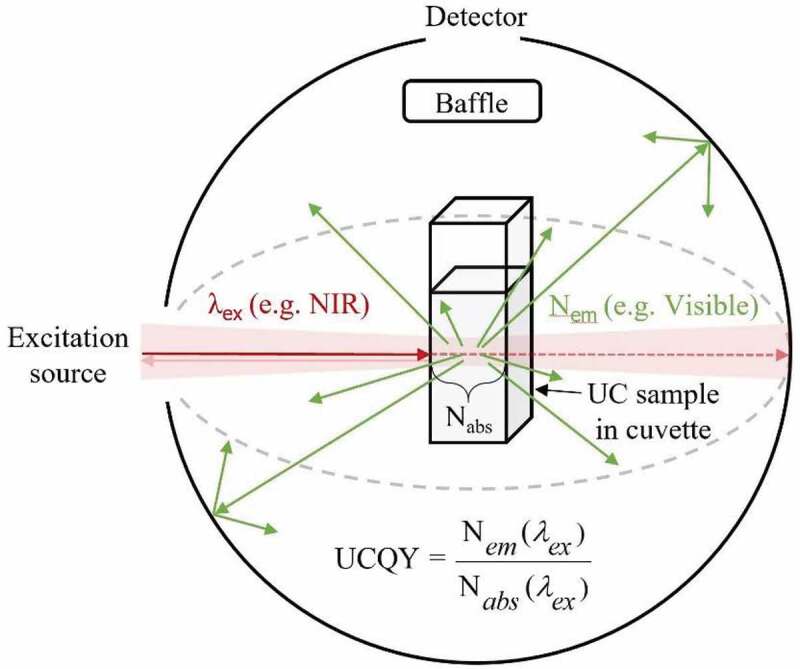
A depiction of an absolute UCQY characterization measurement taking place inside an integrating sphere. Here, near-infrared (NIR) light excites a low-scattering UC sample inside a cuvette, which then emits UC emission in the visible range

The Parker-Rees method determines the PLQY of a given photoluminescence material in relation to a known PLQY standard [69]. Among various limitations, the main impediment arises due to the requirement of the material of interest and the PLQY standard to possess similar absorption and emission properties in comparable wavelength ranges [70–72]. UC materials cannot be characterized in this way as they commonly operate outside the region covered by reliable standards (e.g. >950 nm) [73]. As such, relative UCQYs have still not had their reliability confirmed [15,74], and absolute methods should be sought instead. These measurements do not require a standard and are instead conducted in an integrating sphere so that all the light involved in the measurement can be captured, as depicted in Figure 2. Here, examples are given of excitation and emission light propagating in various directions. The transparency of the arrows indicates the relative intensity of the light, and the dotted arrows indicate an absorption process has taken place. As defined in equation 1, the absolute UCQY is determined by first acquiring the total number of photons emitted (N*_em_*), which is done by measuring the integrated emission intensity (L_Sample_), and dividing its value by the total number of photons absorbed (N*_abs_*). The latter is found by subtracting the intensity of the excitation beam after propagating through the sample under review (E_Sample_) from the intensity of the beam after passing through an equivalent undoped reference sample (E_Reference_), possessing similar scattering properties.

(1) $$UCQY=\frac{{\;}_{Total\;\;number\;\;of\;\;photons\;\;emitted}}{{\;}_{Total\;\;number\;\;of\;\;photons\;\;absorbed}}=\frac{N_{em}(\lambda_{ex})}{N_{abs}(\lambda_{ex})}\;\;=\frac{L_{Sample}}{E_{Reference}-E_{Sample}}$$

The absolute UCQY can also be referred to as the internal UCQY (iUCQY) when an appropriate reference sample is used. Without an adequate reference or when no reference is used, the external UCQY (eUCQY) must be reported. This is an important distinction as the true number of absorbed photons irrespective of scattering effects cannot be determined in this scenario. Overall, iUCQY measurements are ideal for reporting the efficiency of the UC mechanism and eUCQY is more appropriate for characterizing UC devices [68]. Furthermore, the iUCQY has 50% maximum value due to the underlying requirement of at least two photons being absorbed, whilst only one is emitted [68].

It should be stated that the UCQY can also be defined as the integral of the UC photon flux divided by the integral of the absorbed photon flux as an alternative [13,68,75]. Furthermore, the UCQY can additionally be quoted as the radiative portion of the total relaxation rate of a given energy level and determined using photoluminescence decay dynamics and rate equation models [76,77]. As seen in equation 2, the total radiative relaxation rate (A_rad_), which is determined by experimentally measuring the lifetime (τ_exp_), is divided by A_rad_ plus the rate of total non-radiative transition (W_nr_), which are found are both found through Judd-Ofelt analysis [77]. As such, these UCQY values rely on theoretical values and are therefore not as informative as absolute UCQYs.

(2) $$UCQY=\frac{A_{rad}}{A_{rad}+W_{nr}}=\frac{\tau_{\exp}}{\tau_{rad}}$$

### 1.4. Difficulties comparing absolute UCQYs between various materials

The UCQY eventually saturates due to its proportional relationship to the n^th^ power of the excitation power density [78]. At low power densities, ‘n’ is equal to 2, which transitions to 1 as the power density increases [7]. Subsequently, quoting the UCQY at a single excitation power density provides very limited information on the performance potential of the UC material. The normalized UCQY was introduced to make it easier to compare UCQYs acquired at differing power densities. This has units of cm^2^/W since it is obtained by dividing the UCQY by the associated irradiance (I), as shown in equation 3 [29].

(3) $$normalised\;\;UCQY=\frac{UCQY}{I}$$

Although an improvement, normalized UCQYs are not the most ideal solution as the UCQY vs power density slope is still dependent on the irradiance [24]. Furthermore, for an informative study, the UCQY still needs to be obtained over a broad range of excitation power densities, which has time consuming consequences.

Responding to this matter, Andersson-Engels et al. proposed the balancing power density (BPD) [65,79]. This has been used as a tool for obtaining an informative UCQY characterization of UCNPs through a relatively fast approach [79]. The BPD establishes the UCQY when the depopulation of the activator ion’s intermediate state has an equal contribution from the rate of ETU and the linear decay rate. This occurs when the slope between the UCQY and the excitation power density is equal to 1.5, and the irradiance at this point is determined (I_BPD_). Additionally, the UCQY is determined at its saturation point (UCQY_Saturation_). Using these experimental values, the UCQY at various other irradiances can be determined, as described by equation 4.

(4) $$UCQY=\frac{UCQY_{Saturation}\frac{I}{I_{BPD}}}{1+\frac{I}{I_{BPD}}}$$

Despite its benefits, the BPD has been shown to overestimate the UCQY of UCNPs when using moderate excitation power densities (2–11 W/cm^2^) and underestimate it at higher power densities (>11 W/cm^2^), as highlighted by a further piece of work [67]. It has been proposed that thermal effects induced by the excitation laser influence the multiphoton relaxation rate and are the root behind this behavior. As such, the critical power density (CPD) model was then introduced. Here, experimental investigations focus on characterizing the material when these thermal effects are negligible. Equation 5 describes the CPD determination for an ETU system exhibiting two-photon UC.

(5) $$CPD=\;\frac{k_{1^{2}}h\nu }{8k_{ET12}\alpha }$$

Here, ‘*α*’represents the absorption coefficient, ‘*hν*’ is the energy of a photon, and ‘k_1_” is the rate constant of the intermediate state A_1_. The rate at which two ions in A_1_ transfer their energy for one of them to move into state A_e_ and the other to relax to the ground state. Once the CPD is determined, the UCQY at the CPD can be found, which is linked to the saturation UCQY. The CPD UCQY can be determined in a low power density regime, therefore, the saturation UCQY can be theoretically estimated free from thermal influences induced by the excitation laser. Subsequently, this method is also beneficial for groups with limited access to sources that can provide high power density excitation regimes. Overall, the CPD and saturation UCQY can offer an informative and fast characterization of an UC materials efficiency. However, the measurements can still be influenced by various effects that will described later in this work, and the analysis does have limitations due to its reliance on theoretical values.

In addition to the difficulties of comparing UCQYs obtained at different excitation power densities, it should be noted that the UCQY does not have equal contribution across all UC emissions. I.e. a high total UCQY, which involves the integration of all UC emissions, does not guarantee a high UCQY for a specific UC emission and therefore the material may not be appropriate for certain applications. Additional challenges arise due to the absence of a standardized measurement and reporting procedure. This was highlighted by Wisser et al. [80]: *‘The measurement of upconversion (UC) quantum yields is a challenging and nuanced process. Furthermore, small changes in experimental parameters can induce substantial variation in the measured UC efficiency’*. Additionally, in 2017, Kaiser et al. stated [15]: *‘detailed descriptions of the instrument design and actual performance of ΦUC measurements are commonly missing’*. To address these challenges, this work concentrates on highlighting the specific parameters that need to be reported to make these characterizations more comparable. Furthermore, the effects that influence the UCQY measurement are summarized, and information is given regarding when they are significant and how they can be minimized. A literature review is then carried out on previously reported UCQY values, which are categorized based on the experimental parameters reported and significance of effects that are influencing the magnitude of the result. This gives an indication of their reliability and further promotes comparability. Overall, this review is reference point for determining UCQYs with high reliability and comparing new values with previously reported UCQYs. This is the first step towards obtaining UCQY standards, which are defined as UC photoluminescence efficiency values with the highest comparability possible.

## 2. Experimentally characterizing the absolute UCQY

### 2.1. Setups

The experimental setup required for absolute UCQY determinations is very similar to that established for PLQY analysis [81,82]. Subtle differences arise between the two primarily in the measurement methodology and in the range of effects that influence the result, primarily due to the non-linear power dependency of UC materials. In this section, the experimental equipment required for UCQY characterizations will be discussed, followed by notes on what factors should be considered that are not translatable to traditional PLQY analysis.

To begin, the excitation wavelength should be selected based on the type of RE^3+^ dopants and UC mechanism under review. Since RE^3+^ materials with discrete energy levels possess narrow as well as weak absorption cross sections due to the forbidden nature of their parity transitions, their optical properties are frequently characterized at the excitation wavelength with the strongest resonance. For instance, 980 nm is frequently utilized to target the prominent absorption band of the Yb^3+^ ion in around this region. This can be achieved using broadband sources and pulsed laser sources; however, continuous-wave monochromatic laser diodes commonly utilized due to their affordability, the high power densities achievable through focusing, and because the UCQY has been shown to exhibit excitation wavelength dependent properties [83]. These effects will be discussed later in more detail. Due to the sensitivity UC materials towards the excitation power density, these excitation sources must have good stability and temperature control. If these are absent, power variations and wavelength drift effects can be especially problematic. For similar reasons, more affordable setups tend to control the excitation power using optical density (OD) filter in the beams path rather than changing the input current on the laser driver.

Optics are used to align the source with the UC sample, which is placed inside the IS. The integrating sphere possess walls made of material (e.g. Spectralon^TM^) that is highly reflective (>99%) across a broad range of wavelengths. A baffle is places inside the integrating sphere for ensuring all sample emission is isotropic and subject to similar loss effects due to contact with the reflective walls [31]. It accomplishes this by blocking emission light from making direct contact with the measurement detector. The light from the integrating sphere exits through an emission port, where additional optics guide it through an emission monochromator and towards the detector. Photon-multiplying tubes (PMTs) and charge-couple devices (CCDs) detectors are ideal for detecting light in the UV/visible/NIR range. Careful consideration should be given to the detector as it defines the measurement range, resolution, noise level, speed, and cost of the setup. For example, CCDs typically provide greater sensitivity at longer wavelength ranges compared to PMTs [53]. Additional equipment includes the short-pass and long-pass filters required for minimizing second order effects, as well as the bandpass filters used for optimizing the bandwidth of the excitation. Optical density filters are also utilized for controlling the excitation light intensity, in addition to the emission light intensity, to protect the detectors from oversaturation damage. Finally, although used in relative UCQY analysis, polarizers are not needed for absolute methods [73].

### 2.2. Methods

Boyer and van Veggel first reported the absolute iUCQY of colloidally stable NaYF_4_:(2%)Er^3+^,(20%)Yb^3+^ UCNC’s and their bulk phosphor counterparts [84], and the work is regularly cited as an early work for conducting similar measurements. Their technique can be described as a two-measurement method (2 MM), which involves the measurement of the sample emission as well as its absorption. The alternative three-measurement method (3 MM) comprises of an additional step that aims to correct for various scattering effects, as described by Mello et al. [85]. This correction involves measuring the excitation spectra when the samples in under indirect excitation. Although this method has been used in numerous UCQY studies [86–92], direct comparisons of the 2 MM and 3 MM methods were found to yield results with excellent agreement to each other in various UC materials [64,78,93]. Furthermore, as discussed later, all the effects induced by scattering cannot be adequality corrected for in UC materials using this 3 MM method. Overall, the two methods will not be differentiated between during this review.

New methods are also being developed to compensate for limitations associate with UCQY analysis. For example, May et al. describe a characterization technique that uses the Yb^3+^ emission as an internal standard [94]. Interestingly, these efficiency measurements do not require an integrating sphere or a PLQY standard. Instead, it relies on the 1 µm emission decay curve under low pulse energy, an estimation of the 1 µm emission radiative decay rate, and corrected measurement of the UC emission spectra. The method is beneficial as it has greater accessibility and can characterize samples with no measurable absorbance however, it is currently limited to Yb^3+^ doped materials and relies on theoretical values.

### 2.3. Spectrum-related correction and scaling protocols 

Ultimately, the UCQY measurement is informative because it is free from all effects introduced by the measurement equipment. This can be a substantial obstacle for home-built experimental setups because the excitation source photon distribution, FP distance from lens, integrating sphere reflectivity, optical aberrations, monochromator sensitivity, detector sensitivity, and filter transmissions, have magnitudes which vary depending on the wavelength of light. Therefore, spectra correction files need to be implemented that amend for the system response at each wavelength. Furthermore, if two detectors are required to obtain spectra relating to the UCQY calculation, the associated spectra must be scaled to one another. Generally, this involves finding light intensity data of a wavelength common to both detectors.

### 2.4. Beam profile and power density characterization 

Unlike the PLQY studies of materials with linear power decencies, it is crucial that the UCQY value be stated alongside a well-defined excitation power density measurement. This involves characterizing the profile and power of the excitation beam at its focal point (FP). There are various avenues to do this, such as utilizing a commercial beam profiling device (e.g. InGaAs camera [13]), or using the ‘Knife edge’ technique [95]. Other less precise approaches include using burn paper [63], or photography based methods [75]. Since the size of excitation beam diameters are frequently several millimeters in diameter or less, it important that this measurement is well-defined, and the measurement error is stated.

## 3. Considerations for obtaining absolute UCQY of the highest reliability

### 3.1. Influential effects

Unlike photoluminescent materials with a linear power relationship, a variety of additional effects need to be considered when characterising the efficiency of UC materials. The mechanism behind these effects, their influence, and their significance on the observed UCQY will be discussed in the following paragraphs.

#### 3.1.1. Excitation beam profile

**Figure 3.**
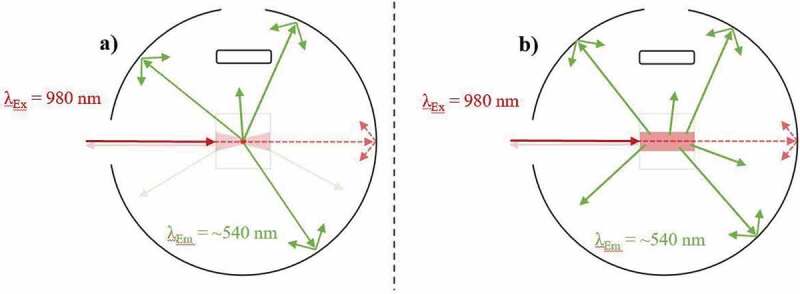
A top-down view of an integrating sphere depicting the effect of the excitation (980 nm) beam profile on the UC emission (~540 nm) in a low-scattering Yb^3+^/Er^3+^-doped sample during UCQY characterisation. Examples are given of excitation possessing a) a near Gaussian beam profile, and b) a near top-hat beam profile

Conflicting UCQY values can be established for identical UC materials despite similar irradiance due to variations in the excitation beam profile. As depicted in Figure 3(a), a Gaussian beam profile concentrates the highest excitation power density towards the focal point (FP) of the beam. As such, variations in the power density are present throughout the sample, which directly relates to differences in the efficiency of local UC mechanisms. The observed UCQY is a representation of combining these local efficiencies together. Alternatively, a top-hat beam profile can provide a more uniform distribution of excitation power density throughout the sample, as shown in Figure 3(b). As such, the local differences in UC efficiency are minimal.

Despite their benefits, top-hat beam profiles are not frequently used for UCQY determination. This is partly due to the inexpensive cost of diode lasers possessing a near Gaussian beam profile, as well as the ease in which high power densities can be obtained by focussing the beam into a small FP [96]. This effect was experimentally investigated by Kaiser et al. [15]. Subsequently, UCQY variations of 20–30% were reported when the authors characterised identical UCNPs using a Gaussian beam profile in comparison to a top-hat beam profile. The authors highlight that deformation of the beam profile also occurs as the excitation propagates through the sample. However, it is stated that the defamation is negligible for samples possessing <10% absorption.

Mousavi et al. proposed a 60% correction for the power density variations that occur when characterising their UCNPs with a non-uniform beam profile [65]. This was achieved by using rate equation analysis to establish a relationship between the excitation power density distribution and the iUCQY [65]. Due to the significance of the effect and magnitude of the correction, it must be considered when comparing the UCQY between samples. Furthermore, the magnitude of the effect will vary depending on how the UC material is embedded into the optical cuvette. Therefore, information on the dimensions and fill factor of the sample inside the optical cuvette should be provided, especially for bulk UC phosphors.

#### 3.1.2. Excitation beam scattering

**Figure 4.**
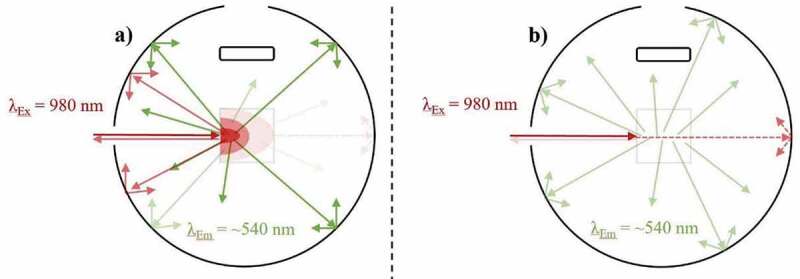
A top-down view of an integrating sphere depicting the effect of excitation (980 nm) scattering on the UC emission (~540 nm) in an Yb^3+^/Er^3+^-doped sample during UCQY characterisation. Examples are given of excitation in a) a high-scattering regime, and b) a low scattering regime

As stated by Würth et al. [74], highly scattering samples decrease the comparability of their PLQY characterisations. However, valuable PLQY data can still be acquired at the time of measurement if a reference sample with adequate scattering properties is used [97]. To increase the measurement comparability in these scenarios, alternative methods have been proposed, which include characterising the sample through indirect excitation [85,97,98]. However, none of these replacement techniques are suitable for characterising UC materials, which demands exact knowledge of the excitation power density that is probing the sample. Furthermore, it is important to note that using an adequate reference sample does not compensate for the effects of scattering when determining the UCQY. For instance, placing an UC sample in a high-scattering scenario (large refractive index (RI) disparity between materials and surrounding media), will increase the material’s UCQY value compared to a highly transparent sample due to increased local power densities induced by scattering [99–101]. An example of this effect is given in Figure 4(a). Here, excitation in a high scattering regime causes higher power densities near the front of the sample. This relates to UC being generated with higher efficiency in this area. However, less excitation light can penetrate through the sample and therefore, weaker UC emission is generated deeper into the sample. Alternatively, as seen in Figure 4(b), the excitation beam can propagate with higher uniformity through a sample in a low low-scattering regime. Subsequently, a lower maximum power density is achieved in comparison, and UC is generated at similar efficiencies throughout the sample. For example, when studying NaYF_4_:(18%)Yb^3+^,(3%)Er^3+^ microscale phosphor, our group determined that the eUCQY (500–700 nm) increased by 403% when the RI disparity was 0.4821 compared to when it was minimal [99]. Further work by our group provided data to show that scattering also caused the iUCQY to saturate at lower excitation power densities and increased the influence of thermal effects inside the sample, which leads to greater UC emission decay rates [100]. Such an observation is important as it suggests that the performance of UC composites is not maximised by matching the RI of the UC material and its encapsulate, despite this being commonly performed [102]. Ultimately, embedding UC phosphor in air creates a high-scattering regime. Although the UCQY data is still beneficial, the efficiency at the given excitation power density is misleading.

Stating an exact threshold particle size for when scattering effects become a significant factor in optical characterisations is challenging. However, it has been estimated that scattering should be considered when particle sizes are 1/10^th^ of the excitation wavelength [74]. This is supported by reports of negligible scattering effects in various studies using 980 nm excitation, which have focussed on UCNPs of various sizes under this threshold [12,84,103]. Furthermore, experimental iUCQY (500–700 nm) data obtained by our group that has shown no relation to the particle concentration of NaYF_4_:(18%)Yb^3+^,(3%)Er^3+^ UCNPs (d = 31.8 ± 9.0 nm) over the range 9.6 to 16.5 mg/mL in water [99]. This characterisation was conducted with 980 nm excitation, therefore, the discussed particle size threshold for scattering was not met. However, it should be noted that even if scattering effects are small, the eUCQY was shown to exhibit a particle concentration dependency since the true number of absorbed photons has not been represented in the measurement due to effects relating to the dispersion solvent. This needs to be considered when comparing the UCQYs of different samples. Finally, it should be noted that some UC materials can be synthesised in the bulk crystalline form, such as BaY_2_F_8_:Er^3+^. This is beneficial as scattering does not represent a limiting factor in these materials, as reported by Fischer et al. [104]. This was noted due to the high similarity between the eUCQY and iUCQY values observed for this material. Such similarities are not observed when studying UC phosphors due to the high significance of scattering.

#### 3.1.3. The primary inner-filter effect

**Figure 5.**
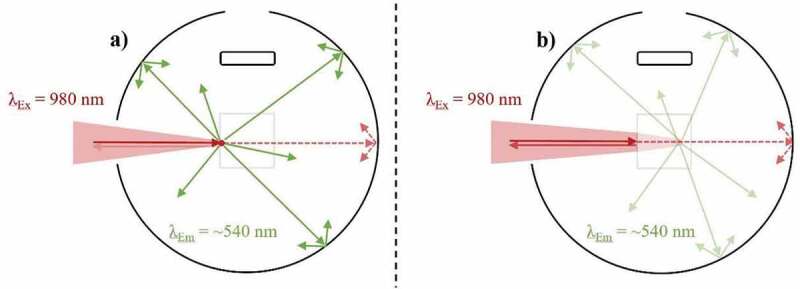
A top-down view of an integrating sphere depicting the primary inner-filter effect on the UC emission (~540 nm) when exciting (980 nm) an Yb^3+^/Er^3+^-doped sample during UCQY characterisation. Examples are given of a) the excitation FP at the front of the sample, and b) the excitation FP deep into the sample

The primary inner-filter effect involves the absorption of excitation light as it propagates through a sample, which results in local power density variations [105–107]. By focussing the laser deep inside a bulk UC sample, the primary inner-filter effect as well as backscattering reduce the maximum power density at its FP. Experimental data has shown that this is a very significant factor to consider when undertaking UCQY analysis. For instance, the iUCQY (500–700 nm) of NaYF_4_:(18%)Yb^3+^,(2%)Er^3+^ phosphor was 94% less when the excitation FP was located 8.4 mm into the phosphor compared to at its front surface [100]. The reduction in power that reaches the FP also leads to UCQY saturation at high excitation power densities as well as decreased thermal effects. The effect of the excitation FP is depicted in Figure 5(a,b), where it is clearly seen that most optimal position is located at the front of the sample as there is minimal power loss. Unfortunately, currently available correction methods for the primary inner-filter effect are not appropriate for UC materials due to their non-linear dependency on the excitation power density [108,109].

#### 3.1.4. The secondary inner-filter effect and sample thickness

**Figure 6.**
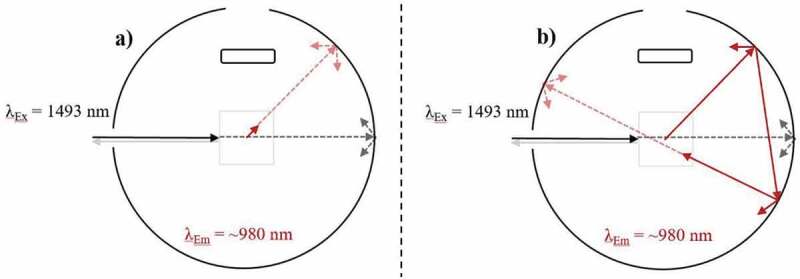
A top-down view of an integrating sphere depicting the secondary inner-filter effect on the UC emission (~540 nm) when exciting (980 nm) an Yb^3+^/Er^3+^-doped sample during UCQY characterisation. Examples are given of a) self-absorption of the UC emission prior to it leaving the sample, and b) self-absorption of the UC emission after is has the left the sample

The secondary inner-filter effect, or emission self-absorption, is a common impedance on the PLQY analysis of organic dyes. This is understandable due to the significant overlap between the absorption and emission bands possessed by these materials. However, these effects are commonly overlooked when characterizing UC materials due to the larger anti-Stokes gap between the UC emission bands and the primary absorption band [74]. However, various studies have noted that these effects should still be considered [32,110]. For example, by exciting β-NaYF_4_:Er^3+^ at 1493 nm, the sample exhibits UC emission in the range ~950-1050 nm. This directly overlaps with one of the samples absorption bands in the same region and therefore, self-absorption of the UC emission can occur, as investigated experimentally by Boccolini et al. [111]. Figure 6(a) depicts self-absorption of the UC emission prior to leaving the sample, and Figure 6(b) shows self-absorption of the UC emission after it re-enters the sample due to reflection from the walls of the IS. Experimental findings by Boccolini et al. showed that the UCQY (900–1100 nm) of BaY_2_F_8_:(30%)Er^3+^ UC substrates was limited due to the effects of self-absorption [111]. A correction was proposed that involved normalizing each emission spectra to intensity obtained at 1020 nm. At this wavelength, the absorption coefficient is minimal, and the emission magnitude is high, therefore the effects of self-absorption are negligible. By comparing normalized spectra taken both inside and outside the IS, the total effects of self-absorption were determined to cause emission losses of 47%, 30%, and 51% in samples of 10%, 20%, and 30% Er^3+^-doping, respectively. A supporting theoretical investigation by the same author indicated that the relationship between sample thickness and the molar concentration of Er^3+^ needed to be optimized to minimize these self-absorption effects [112]. The magnitude of this effect in other UC materials depends on this relationship as well as the magnitude and overlap of the absorption and emission ranges exhibited by the material.

#### 3.1.5. Absorbance and particle concentration

Due to the low number of dopants in the excitation path, small absorptions are usually recorded when studying UCNPs. This is a challenge for absolute UCQY measurements due to the limited precision achievable when utilizing an IS. Furthermore, small excitation fluctuations can lead to a prominent source of error, even when ultrasensitive detectors are used [24]. However, if absorption values are too high, inner-filter effects, particle concentration related backscattering effects, and indirect excitation effects, can become obtrusive. In response to this, an IUPAC technical report on traditional PLQY analysis suggested that a sample absorbance (A) of <0.1 be used for analysis and ensure the minimisation of these effects [72]. In terms of the UCQY, reports have suggested that an absorption of at least 4–5% be used when studying UCNPs to keep the influence of these effects low whilst still maintaining a sufficient signal-to-noise ratio [65]. Other studies have suggested similar absorption values of 3–6% during their analysis, which, in this example was achieved using a UCNP particle concentration of 40 mg/mL [78]. Kaiser et al. prepared their UCNPs with an A = ~0.02 at the excitation wavelength [15]. In doing so, the group reported UCQY data that was comparable to theoretical predictions, which indicated that the influence of external effects was small, and that the absorbance threshold suggested was beneficial for obtaining reliable UCQYs.

**Figure 7.**
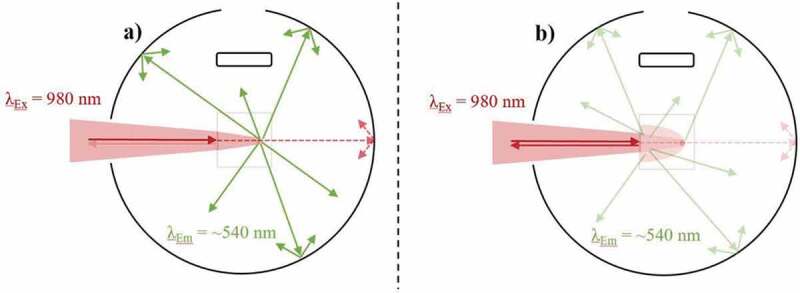
A top-down view of an integrating sphere depicting the effect of particle concentration on the UC emission (~540 nm) when exciting (980 nm) an Yb^3+^/Er^3+^-doped sample during UCQY characterisation. Examples are given of exciting a) a low particle concentration sample, and b) a high particle concentration sample

In terms of encapsulated sub-micron particles that have an absorbance far greater than this threshold, our group has shown that the particle concentration does show a relationship to the UCQY [99]. Here, the eUCQY (500–700 nm) of NaYF_4_:(20%)Yb^3+^,(3%)Er^3+^ particles encapsulated in PDMS became limited at high particle concentration due to increased backscattering and inner-filter effects. A comparison between the characterisation of samples with low and high particle concentrations is given in Figure 7(a,b).

#### 3.1.6. Reference material

**Figure 8.**
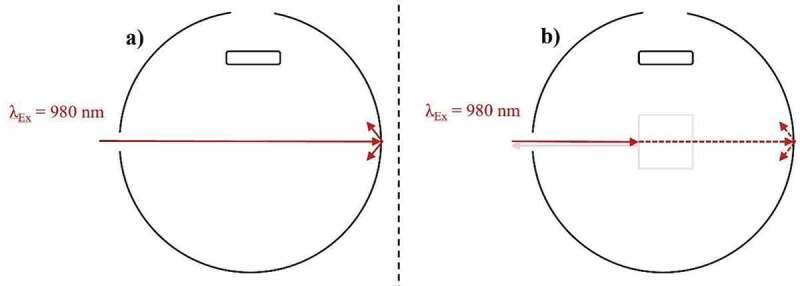
A top-down view of an integrating sphere depicting the effect of the reference sample when exciting (980 nm) an UC sample during UCQY characterisation. Examples are given of a) using no references sample, and b) using an undoped reference sample with high transparency

As stated, an appropriate reference sample is required for iUCQY analysis. This is sometimes bypassed in UCNP analysis by using a reference sample of pure dispersion solvent [80,113–117]. This is acceptable since the UCNPs under review do not meet the particle size dependent size threshold for scattering (d < 10% of excitation λ), and the effects of the dispersion solvent are still represented in the absorption measurement. Furthermore, experimental UCNP observations concluded no discernible UCQY difference between using an undoped NP reference sample or one consisting of pure dispersion solvent at this size scale [84]. However, it should be repeated that even when this size threshold is met, discrepancies between the iUCQY and eUCQY arise because of effects relating to the dispersion solvent in the absorption measurement. This is represented in Figure 8(a,b). Here the effects on the excitation beam when the integrating sphere is empty are compared to when it propagates through an undoped reference sample with a high transparency. Despite the transparency of the later, small absorption and backscattering effects are still present, which influence both the absorption and UCQY measurement.

#### 3.1.7. Dispersion solvent and encapsulating media

As stated, quenching effects that arise from the solvent that UCNPs are dispersed in due to multiphoton nonradiative relaxation [118]. Therefore, significant UCQY increases can be observed in these materials if they dispersed in highly non-polar solvents. Additionally, passivation layers and shells structures can be synthesised into their structure to reduce the influence of these effects [119–128]. To ensure that high levels of excitation power density reach the UCNPs, dispersion solvents with high transmission in the UV/visible/NIR range and high purity are commonly selected [72]. When characterising UCNPs dispersed in a material that partially absorbs the excitation light, it has been suggested that correction be applied to compensate for the reduced power that reaches the UC material. A method for estimating this power reduction has been previously established [129]. Thermal effects can also be induced due to the absorption of the media surrounding UCNPs and these must also be considered as discussed in the next sub-section [129].

#### 3.1.8. Thermal effects

PLQY standards are reported alongside their temperature due to the significance of thermal quenching by multiphonon nonradiative relaxation [130]. In a similar manner, this is also a requirement when reporting UCQY standards. However, additional consideration must also be given to variations in local temperature that are induced by the excitation beam, as discussed earlier. At a sufficient excitation power density, the UC emission becomes intrinsically coupled to the local temperature inside the sample [131]. For instance, a 24% decrease in UC emission intensity has been observed in β-NaYF_4_:(18%)Yb^3+^,(2%)Er^3+^ after the sample temperature increased by 60 K under 250 W/cm^2^ excitation for 1 min [132]. This makes it challenging to determine the true UCQY saturation point in bulk UC materials, as it commonly occurs in a high power density regime, where these effects are very prominent [74]. In these environments, the UCQY has been observed to decrease as the power density increases due to these effects [67]. The threshold for thermal effects becoming influential in bulk UC phosphors has been estimated at excitation power densities >10 W/cm^2^ [67]. However, the sample’s composition, size, structure, and scattering regime, will all be influential factors on the exact threshold [100]. To address these effects, some groups have implemented limits to the sample’s excitation exposure time as well as breaks between excitation periods [15,96,129]. However, due to the small timescale involved (<30 ms) [132], these will likely have minimal benefit. As previously stated, the CPD is currently the most useful method for obtaining relevant UCQY data irrespective of laser induced thermal effects [67]. However, readers should be away of their dependence on theoretical values.

With respect to colloidally stable UCNPs, Homann et al. state the heat induced by excitation dissipates more easily since the particle are dispersed [115]. Therefore, higher excitation power densities can be investigated without significant influence of these effects. Experimental data supports this, as research has shown that no significant heating effects occurred when studying them in NaYF_4_:(18%)Yb^3+^,(2%)Er^3+^ UCNPs dispersed in toluene [132].

#### 3.1.9 Integrating sphere and sample position

**Figure 9.**
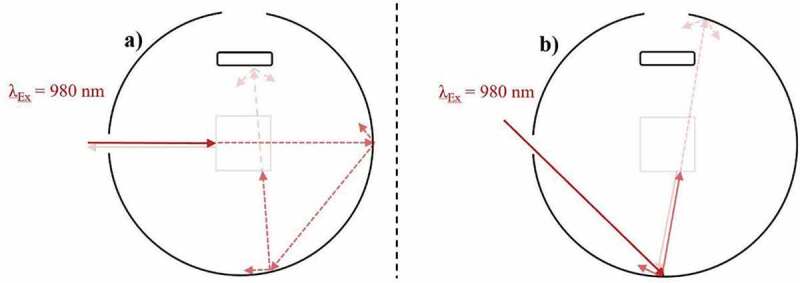
A top-down view of an integrating sphere depicting the effect of indirect excitation when exciting (980 nm) an UC sample during UCQY characterisation. Examples are given of indirect excitation after a) reflection off the walls of the IS, and b) beam misalignment

The integrating sphere model should not be overlooked when attempting to acquire measurements of the highest reliability. As stated, a baffle is a fundamental requirement for the IS. Furthermore, the size of the integrating sphere should be sufficient to minimize the effects of indirect excitation. As shown in Figure 9(a), excitation light that has propagating through the sample can reflect from the walls of the integrating sphere and enter the sample again. This increases the excitation photon pathlength and the probability of an UC process occurring, which skews the observed UCQY measurement. Integrating spheres with inner diameters such as ~150 mm have been used to minimize this effect and produce results of a high standard [84]. Indirect excitation can also occur if the beam is misaligned with the sample, as depicted in Figure 9(b). Care should be taken to avoid this scenario as it leads to uncertainty in the excitation power density and beam profile that interacts with the sample. The third consideration to make when selecting an integrating sphere is the size and number of ports it possesses. Reducing these parameters minimizes light loss effects, which involves emission and excitation light never reaching the detector [98]. This effect is magnified when studying high scattering materials due to the heightened levels of backscattering [133,134].

**Figure 10.**
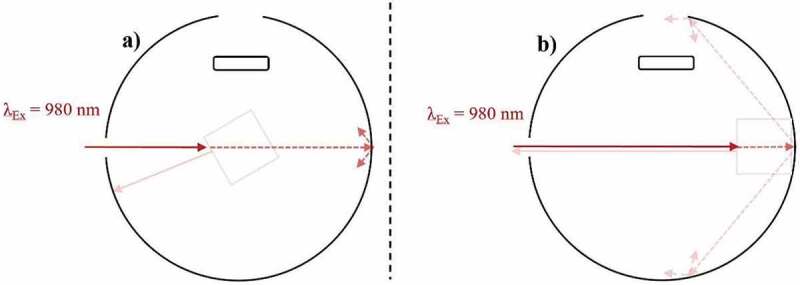
A top-down view of an integrating sphere depicting the effect of sample position when exciting (980 nm) an UC sample during UCQY characterisation. Examples are given of exciting a sample a) placed on the wall of the IS, and b) placed at a non-perpendicular angle to the excitation beam

When the integrating sphere cannot be altered, researchers frequently reduce the light loss effect by placing the sample at a non-perpendicular angle to the excitation beam. This procedure reduces the amount of backscattered excitation that exists through the integrating sphere’s excitation port and is a technique commonly used in traditional PLQY analysis [15,83]. However, as seen in Figure 10(a), this changes the excitation photon path length inside the sample. Given the evidence already presented, such a change in the excitation power density distribution can have significant effects on the emission of UC materials. Furthermore, if probing a high scattering material, significant beam distortion effects can arise due to the angle of incidence [100]. This will affect the maximum power density experienced by the sample as well as the magnitude of the primary inner-filter effect, both of which can have substantial consequences. Ideally, these uncertainties should be minimized by maintaining an excitation beam that is perpendicular to the UC sample. The resulting light leakage effects can by reduced through alternative means such as decreasing the size and number of ports on the IS. For instance, an optical fiber coupled integrating sphere can provide such benefits.

The placement of the UC sample inside the integrating sphere should also be considered. The sample is ideally placed in a position that minimizes influential effects such as indirect excitation, which is usually at the center of the IS. As shown in Figure 10(b), when the sample is placed on the wall of the IS, the probability of excitation light propagating through the samples, reflecting from the wall, and reentering the sample is increased. This is less of a concern in traditional PLQY measurements as the increased absorption events are linearly proportional to greater emission events. However, this should be avoided when studying UC materials due to their non-linear power dependence. It is acknowledged that sometimes the samples position is restricted by the model of the integrating sphere and the design of its sample holder. Therefore, in these scenarios it should be clearly stated that this effect is potentially influencing the reported UCQY. As a final note on this topic, the sample holder itself should be able to ensure similar sample positions for every spectrum taken. Some holders do not secure the sample in a certain position, which can increase variations in the excitation FPs position in the sample and therefore, increase the UCQY measurement error [13,96].

#### 3.1.10. Cuvette type and sample dimensions

**Figure 11.**
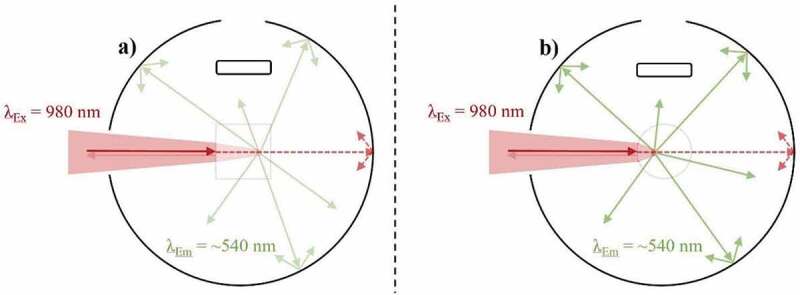
A top-down view of an integrating sphere depicting the effect of cuvette type on the UC emission (~540 nm) when exciting (980 nm) an Yb^3+^/Er^3+^-doped sample during UCQY characterisation. Examples are given of exciting an UC sample embedded in a) a square cuvette, and b) a circular vial

Previous reviews on the PLQY condone the use of a standard 10 × 10 mm square quartz cuvette, in order to minimize optical aberrations and establish uniformity in the procedure [73]. Due to the power sensitivity of UC materials, the cuvette becomes an even greater concern when conducting UCQY characterizations. For instance, our group provided theoretical evidence that circular vials can distort the excitation beam through a lensing effect. This decreased the excitation FP and moved it closer to the front of the sample, both of which are beneficial to the UC mechanism. Experimentally, this led to a 27% increase in the UCQY of NaYF_4_:(18)Yb^3+^,(2%)Er^3+^ phosphor in comparison to when it was embedded in a square quartz cuvette [100]. These effects are shown in Figure 11(a,b), which compare the two vials. Although greater UCQY can be achieved in the circular vial, there is uncertainty in the position and size of the excitation FP. Therefore, standard square cuvettes are recommended. However, readers should be reminded that the sample thickness inside the cuvette is also an influential factor when studying bulk materials, as highlighted earlier.

#### 3.1.11. Sample emission around the excitation wavelength

**Figure 12.**
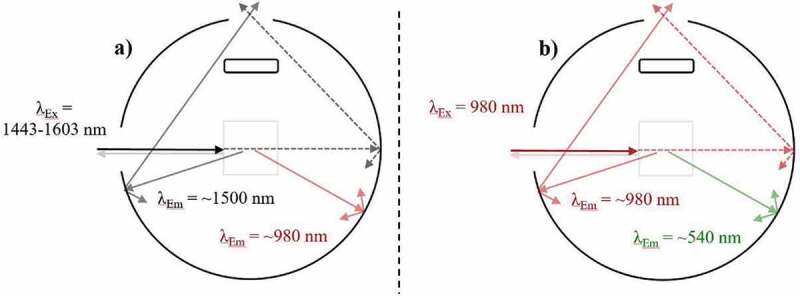
A top-down view of an integrating sphere depicting the effect of sample emission around the excitation wavelength in an Yb^3+^/Er^3+^-doped sample during UCQY characterisation. Examples are given of a) broadband excitation ~1450-1600 nm and emission ~1500 nm, and b) excitation ~980 nm and emission ~980 nm

The UCQY has also been shown to be linked to the integration range used during analysis. This relationship arises due to sample emission that occurs around the excitation wavelength. Variations occur in the UC materials ability to upconvert the absorbed photons, which leads to a re-emission effect that is associated with the composition of the material, as described by MacDougal et al. [83]. The authors showed this effect in β-NaYF_4_:Er^3^ using broadband excitation (~1443-1603 nm), as depicted in Figure 12(a). They also proposed a detailed correction method that consists of scaling the obtained spectra to determine a corrected fraction of absorbed photons. The scaling factor is obtained by measuring the emission at a wavelength where the overlap between the emission and excitation is minimal. Using the corrected absorption measurement, a better estimate of the true UCQY can be obtained. In this example, the iUCQY (~900-1050 nm) was amended from ~24% to ~9% in β-NaYF_4_:(10%)Er^3+^, and from ~6.5% to ~6% in β-NaYF_4_:(40%)Er^3+^ due to this correction.

Furthermore, evidence supplied by Fischer et al. when studying UCNPs with a range of compositions suggested similar issues arise when exciting Yb^3+^/Er^3+^-doped materials with 980 nm excitation [135]. This was due to the Er^3+^ (^4^I_11/2_→^4^I_15/2_) and Yb^3+^ (^2^F_5/2_→^2^F^7/2^) transitions around the 980 nm range and it led to misleading absorption results, as depicted in Figure 12(b). The correction used comprised of exciting the samples at 985 nm and then at 965 nm. The resulting emission spectra obtained could then be stitched together and taken away from the excitation spectra to acquire an amended absorption value. This group used a similar correction in another study where it resulted in the iUCQY (500–700 nm) decreasing from ~6.5% to ~4.0% in β-NaYF_4_:Yb^3+^,Er^3+^@β-NaLuF_4_ core/shell UCNCs [17]. It was highlighted that the emission around 980 nm was prominent in UCNCs with thick shells but negligible in samples with thin shells or where they were absent. Finally, Kaiser et al. reported that this effect was also present in β-NaYF_4_:(21.4%)Yb^3+^,(2.2%)Er^3+^ UCµP, where it resulted in eUCQY (370–890 nm) overestimations of 14% at 0.2 W/cm^2^ and 3% at 100 W/cm^2^ [15]. Overall, the magnitude of the effect appears to be reliant on the UC materials composition, its ability to upconvert all the incident excitation photons, and the excitation conditions.

#### 3.1.12. Measurement sequence, errors, and repeatability

The measurement sequence of spectra required for the UCQY determination should also be considered [96]. Issues can arise due to the laser instability issues that can evolve over time. For instance, wavelength drift, peak distortion, and power density variations can become a significant source of error if the UC sample and the reference sample spectra are not characterised consecutively, within a short period of time. Delays between the measurements occur due to the scanning technology of many spectrometers as well as the necessity to introduce or remove filters between characterising the emission and excitation beam spectra. Additionally, it is less time consuming to measure one sample over a range of excitation power densities before removing it from the integrating sphere chamber, and therefore the idealised procedure is not always adhered to. In addition to this point, a reliable method for quickly determining the excitation power density should be built into the setup to ensure its stability.

Measuring photon counts to determine the absolute UCQY is a highly sensitive procedure and as such, it is important to report the associated measurement error. This error is associated with the sensitivity of all the optical equipment involved in the measurement. This includes the stability of the excitation source, response of the emission monochromator and gratings, and the linearity of the photodetector as well as its noise level. By addressing these factors, this error can be reduced, and the precision of the measurements can be increased. Further precision can be obtained by optimising the measurement scan rate, dwell time, step size, and ensuring similar sample positions for each spectra analysis [96].

### 3.2. Summary of influential effects and suggested UCQY methodology, nomenclature, and reporting architecture

**Figure 13.**
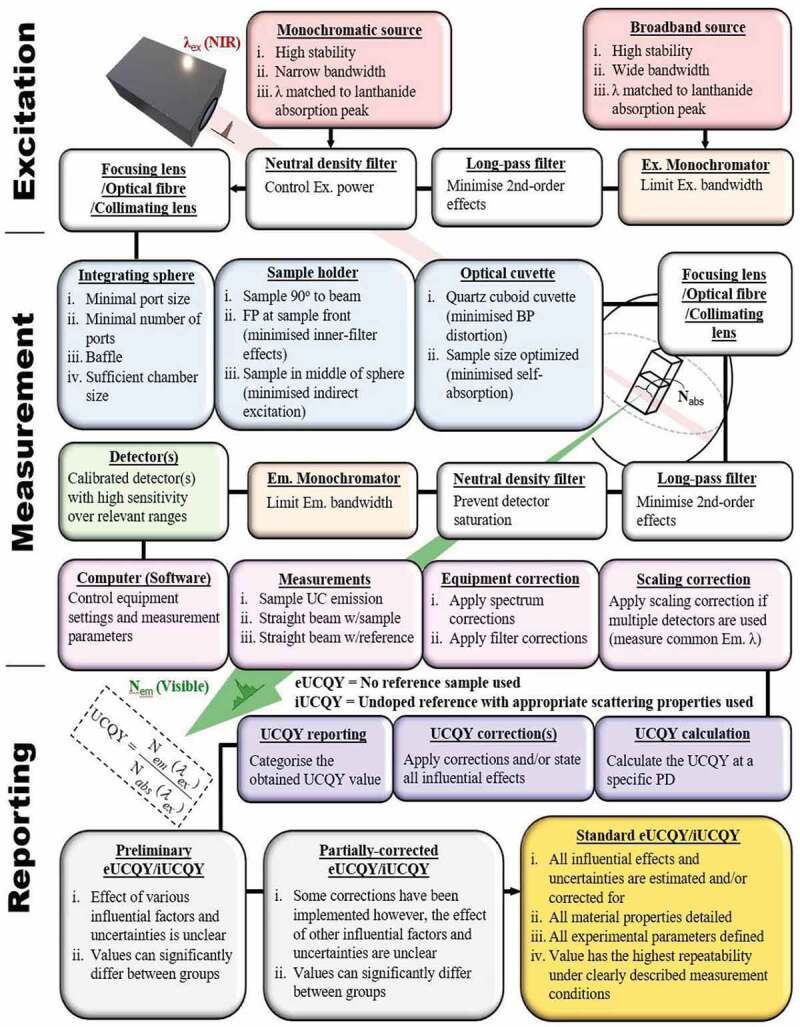
A generalised experimental method and reporting architecture for obtaining UCQY values with high comparability, which is depicted as a flow chart

The various effects that have been discussed are summarized in Table 1. Here, they are assigned alphabetical labels so that they can be easily attributed to reported UCQY values in the next section. Additionally, information on how to bypass or minimize their influence is given and examples indicating their significance are provided. A clear path for obtaining the most comparable values is provided in the flow chart shown in Figure 13. The generalized UCQY methodology architecture presented is also useful for highlighting which experimental parameters are important to consider before characterization and report afterwards. To make such a flow chart, it was necessary to define new UCQY nomenclature. If a value is obtained under the influence of various effects described in Table 1, it is referred to as the preliminary UCQY. Furthermore, if various procedures have corrected for some of these effects, a partially-corrected UCQY term is stated. Ultimately, the standard UCQY is the most important term, and it is reserved for values that are independent of all influential effects. This can be either through their minimization, bypassing them, or reliably correcting for them. Resultantly, these values are the most desired, have the highest comparability. Although laborious, establishing these UCQY standard across a broad range of excitation power densities until saturation for various UC materials is key for comparative assessments and advancing the field further [15]. However, it is acknowledged that since these saturation effects can occur at very high excitation power densities (e.g. ~4400 W/cm^2^ [78]), this is a cumbersome task and adds to equipment availability issues.

**Table 1.** A summarised list of the effects that influence the UCQY measurement reliability is given along with an associated alphabetical label, minimization or bypass method, and an example(s) of its significance as reported in the literature

Effects and uncertainties that influence UCQY (Label)	Issue	Response
Excitation beam profile (A)	Power density variations throughout sample [15,65].	i) Use a top-hat beam profile to minimise this effect. ii) Or correct for the excitation distribution due to the beam profile used.
Excitation scattering and beam profile distortion (B)	Power density variations throughout the sample [15,74,99,100].	i) Scattering minimized if d < 1/10 of Ex. λ. ii) Beam profile distortion minimised if particle concentration is small and sample Abs < 10%.
Primary inner-filter effect when measuring with a focused beam (C)	Backscattering and absorption reduce maximum power density at the beams FP [100].	i) Position FP at front of sample to reduce power loss.
Secondary inner-filter effect (D)	UC emission is self-absorbed by the sample before reaching the detector [32,111,112].	i) Reduced if sample A < 0.1 ii) Optimize relationship between sample thickness and molar concentration of lanthanide dopants.
Media surrounding UC material partially absorbs excitation light (E)	The power density reaching the UC material is reduced and additional thermal effect can be induced [68,129].	i) Estimate excitation power loss. ii) Estimate magnitude of induced thermal effects.
Thermal effects (F)	Environmental and excitation induce thermal effects that can reduce sample emission via multiphonon nonradiative relaxation [67,129,132].	i) Report measurements at room temperature. ii) Minimized when characterizing bulk sample at power densities >10 W/cm^2^. iv) Minimized if particles are colloidally dispersed.
Non-ideal sample position or IS (G)	Effects such as indirect excitation can become more prominent if the sample position is not ideal or an inadequate integrated sphere is used [7].	i) Minimise indirect excitation by placing the sample at the center of the IS. ii) Place sample perpendicular to excitation beam to minimize beam distortion effects. iii) The integrating sphere must be sufficiently large and possess a baffle.
Sample emission interferes with absorption characterisation (H)	Absorption underestimations due to emission photons with similar wavelengths to the excitation being recorded by the detector [17,135].	Measure emission spectra round the excitation wavelength and subtract it from the excitation spectra to obtain a corrected absorption value.
Excitation power density measurement error not reported/power density only reported in graphical format (I)	Error influenced by the precision in measuring the power density at the beams FP [91].	Report power densities and statistical errors in text.
UCQY measurement error not reported/UCQYs only presented in graphical format (J)	Error influenced by equipment sensitivity, laser stability, and variations in sample position between measurements [13].	Report UCQYs and statistical errors in text.
UCQY determined through non-absolute measurement methods (K)	Relative UCQY measurements currently have reliability issues [73].	Use absolute UCQY methods.

## 4. Reviewing the UCQY reporting and measurement techniques in the literature

A broad range of the UCQY literature is reported and categorized in this section using the alphabetical labels outline in Table 1. In doing so, the reliability of each value is indicated, and values obtained under the influence of similar effects can be better compared. The analysis is also useful for highlighting which materials can be used to exploit the highest efficiencies of certain UC emissions and which are ideal for specific applications. An asterisk (*) indicates information that has been logically assumed by our group based on the limited information provided in the literature (e.g. UC emission integration ranges). Alternatively, in instances of limited information and a logical assumption cannot be made, a question mark symbol (?) is used to suggest that a certain effect could be influential (e.g. excitation FP position optimization when characterizing bulk UC samples).

Scattering affects were assumed to be significant if dry powder has been characterized, irrespective of particle size. Additionally, these powders were assumed to possess A > 0.1 and subsequently were influences by UC emission self-absorption. Furthermore, if the exact UCQY methodology was not clearly explained or if an appropriate reference sample was not mentioned, the results was categorized as an eUCQY measurement. Finally, thermal effects in bulk materials were considered significant if the power density exceeded 10 W/cm^2^ [67].

Various UCQYs acquired through non-absolute methods are summarised in Table S1 of the Supporting Information. This includes values obtained using power ratio methods, lifetime based measurements, and internal standard methods [94]. However, the literature review primarily concentrates on UC materials characterised through absolute UCQY methods. Some studies only present the UCQY and power density data through a graphical format, which can be difficult to review precisely due to the logarithmic format commonly used. Where a reasonable estimate cannot be interpreted reliable from the graphs, the UCQYs were omitted from the review. Moreover, values highlighted by the authors or shown to be maximal after optimising the materials dopant composition were favoured over others. Overall, all UCQY values selected for representation were chosen under the discretion of the authors of this work with the intent to achieve a broad understanding of the UCQY reporting techniques used.

### 4.1. UCQY measurements of micro-scale UC materials

The reported values and measurement parameters are initially reviewed for a variety of microscale UC materials, as can be seen in Table 2. Due to their size and the variety of influential effects that comes with it, these materials have the most issues in terms of UCQY reliability. Despite this, these effects are rarely estimated or corrected for. In fact, they are hardly mentioned. This relates to the second problem which involves the range of underreported experimental parameters. The non-standardized reporting structure has led to large variations in the parameters that have been reported such as the UCQY methodology (eUCQY or iUCQY) used, the excitation power density and beam profile, the position of the excitation FP, the optical cuvette type, and a breakdown of which effects are influencing the reported value. Additionally, sometimes critical information about the samples under investigation is absent such as its exact composition, absorbance, and particle size. Clarity regarding the UC emission being studied can also be improved in a variety of cases. For instance, the term ‘Total UCQY’ can be misleading as frequently it does not encompass all UC emissions produced by the sample. Here, the following abbreviations are used; Dim: dimensions, Con: concentration, Abs: absorbance, V: volume, D: diameter, W; width, L: length, T: thickness, LD: long diagonal, SD: short diagonal, PL: path length, Ex. λ: excitation wavelength, BP: beam profile, PD: power density, Em. λ: emission wavelength, Gauss: Gaussian or near Gaussian BP, TH: top-hat or near top-hat BP, Ellip: Elliptical BP, CD: colloidally dispersed, Ad.: Influential effects and uncertainties that have been addressed, and PS REF: pure solvent reference sample.

**Table 2.** Reviewing the reported values and measurement parameters for UCQY characterisations of microscale UC materials

**Sample**	**Type**	**Particle Size** **[shell]** **(Shape)**	**Dim**. **(Con.)**	**Abs.**	**Ex. λ** **[nm]** **(BP)**	**PD** **[W/cm^2^]**	**UCQY [%](Em. λ [nm])**	**N. UCQY** **[cm^2^/W]**	**Effects**	**Ad.**	**Category** **[Ref.]**
β-NaYF_4_: (21.4%)Yb^3+^,(2.2%)Er^3+^	Powder	3 µm (*Varied)	ound cuvette V: 2 mm³, D: 5 mm, PL: 0.1 mm	*>0.1	976 (TH)	20 (Stability < 0.1 %) 130 (Stability < 0.1 %)	10.3 (370-890) 0.92 (833-880) 0.13 (783-833) 6.4 (630-685) 2.4 (510-570) 0.17 (394-430) 8.3 (370-890) 0.52 (833-880) 0.11 (783-833) 5.5 (630-685) 1.8 (510-570) 0.19 (394-430)	0.34 0.031 4.3 x10^−3^ 0.21 0.08 5.7 x10^−3^ 0.064 0.004 8.5 x10^−4^ 0.042 0.014 1.5 x10^−3^	B, D?, F, H?, J	-	eUCQY [15]
NaYF_4_: (20%)Yb^3+^,(2%)Er^3+^	Powder	4 ± 1 µm (*Varied)	-	*>0.1	977 (*Gauss)	0.5 ± 0.01 3.8 ± 0.07 22 ± 0.44	0.21 ± 0.3 (350-900) 0.16 ± 0.02 (*750-900) 0.024 ± 0.004 (*600-700) 0.03 ± 0.004 (*500-600) 1.67 ± 0.3 (350-900) 0.86 ± 0.1 (*750-900) 0.43 ± 0.06 (*600-700) 0.37 ± 0.05 (*500-600) 0.014 ± 0.002 (*350-450) 7.8 ± 1.2 (350-900) 3.53 ± 0.50 (*750-900) 2.5 ± 0.4 (*600-700) 1.66 ± 0.2 (*500-600) 0.11 ± 0.02 (*350-450)	0.42 0.32 0.048 0.06 0.44 0.23 0.11 0.097 3.7 x10^−3^ 0.36 0.16 0.11 0.076 5 x10^−3^	A, B, C?, D?, F, G, H?	C?	eUCQY [136]
NaYF_4_: (20%)Yb^3+^,(3%)Er^3+^	Powder	1 µm (*Varied)	L: 8 x 8 mm T: <1 mm	*>0.1	970 (*Gauss)	21	6.9 ±0.5 (500 – 700)	0.33	A, B, C?, D?, F, H?, I	C?	eUCQY [91]
β-NaYF_4_: Yb^3+^,Er^3+^	Powder (Anneal 500 ^o^C)	2.3 µm (Deteriorated hexagonal)	-	*>0.1	980 (*Gauss)	50	2.7 ± 0.5 (500-700) 1.5 ± 0.3 (625-700) 1.3 ± 0.3 (500-575)	0.054 0.03 0.026	A, B, C?, D?, F, H?, I	C?	iUCQY [137]
β-NaYF_4_: Yb^3+^,Er^3+^	Powder (Anneal 400 ^o^C)	700 nm (Deteriorated hexagonal)	-	*>0.1	980 (*Gauss	50	4.8 ± 1 (500-700) 3 ± 0.6 (625-700) 1.8 ± 0.4 (500-575)	0.096 0.06 0.036	A, B, C?, D?, F, H?, I	C?	iUCQY [137]
β-NaYF_4_: Yb^3+^,Er^3+^	Powder (Anneal 350 ^o^C)	300 nm (Cubic)	-	*>0.1	980 (*Gauss)	50	2.5 ± 0.5 (500-700) 1.6 ± 0.3 (625-700) 0.9 ± 0.2 (500-575)	0.05 0.032 0.018	A, B, C?, D?, F, H?, I	C?	iUCQY [137]
β-NaYF_4_: (20%)Yb^3+^,(2%)Er^3+^	Powder	*<5 µm (Micro-cylindrical)	-	*>0.1	976 / 935 (*Gauss)	60	4.0 (*510-680) 1.7 (*510-580) 2.3 (*630-680)	0.067 0.028 0.038	A, B, C?, D?, F, H?, I, J	C?	eUCQY [138]
β-NaYF_4_: (20%)Yb^3+^,(2%)Er^3+^	Powder	>>100 nm (*Varied)	-	*>0.1	980 (*TH, D: 1 mm)	20	3.0 ± 0.3 (*500-600)	0.15	B, C?, D?, F, H?, I	C?	iUCQY [84]
NaYF_4_: (20%)Yb^3+^,(3%)Er^3+^	Powder	~1 µm (*Varied)	L: 8 x 8 mm T: < 1mm	*>0.1	970 (*Gauss)	21	6.9 ±0.5 (500 – 700)	0.33	A, B, C?, D?, F, H?, I	C?	eUCQY [91]
NaYF_4_: (20%)Yb^3+^,(2%)Er^3+^	Powder	W: 300-500, L: 1900-2200 nm (Cubic + hexagonal)	-	*>0.1	980	-	0.196 (*~500-538) 0.338 (*~538-580) 1.445 (*~620-700) 1.980 (*~500-700)	-	A, B, C?, D?, F?, G, H?, I,	C?	eUCQY [139]
NaYF_4_: (20%)Yb^3+^,(2%)Er^3+^,(30%)Gd^3+^	Powder	W: 200-450, L: 700-1000 nm (Hexagonal)	-	*>0.1	980	-	0.177 (*~500-538) 0.379 (*~538-580) 0.991 (*~620-700) 1.548 (*~500-700)	-	A, B, C?, D?, F?, G, H?, I,	C?	eUCQY [139]
NaYF_4_: (20%)Yb^3+^,(3%)Er^3+^	Powder	~4.2 ± 2 µm (*Varied)	-	*>0.1	980	1	0.0475 (*500-600) 0.0159 (*600-700)	0.0475 0.0159	A, B, C?, D?, H?, I, J	C?	eUCQY [99]
NaYF_4_: (20%)Yb^3+^,(2%)Er^3+^	Powder	*~500 nm (Hexagonal + cubic)	-	*>0.1	972 (*Gauss)	-	~0.03(510-565nm) *~0.04(640-680nm) *~0.07 (510-680nm)	-	A, B, C?, D?, F?, H?, I, J	C?	eUCQY [92]
NaYF_4_: (20%)Yb^3+^,(2%)Er^3+^	Powder	*~4500 nm (Hexagonal + cubic)	-	*>0.1	972 (*Gauss)	-	*~4 (510-565nm) *~9 (640-680nm) *~13 (510-680nm)	-	A, B, C?, D?, F?, H?, I, J	C?	eUCQY [92]
β-NaGdF_4_: Yb^3+^,Er^3+^	Powder	-(*Varied)	-	*>0.1	980 (*Gauss)	*100	2.4 (*400-750)	0.024	A, B, C?, D?, F, H?, I	C?	eUCQY [140]
β-NaYF_4_: (18%)Yb^3+^,(2%)Er^3+^	Powder	-(*Varied)	Epoxy encapsulated 1×1 mm^2^ glass capillary	*>0.1	980 (*Ellip)	0.7 ± 0.1 CPD	0.8 (510−542) 0.4 (820−870) 7.0 (Theoretical saturation)	1.14 0.57-	A, B, C?, D?, H?, J	C?	eUCQY [67]
-NaYF_4_: (18%)Yb^3+^,(2%)Er^3+^	Powder (In air)	Several µm (*Varied)	T: 10 mm	>0.1	980 (*Gauss)	17 ± 3 0.17 ± 0.03 0.025 ± 0.00	5.1 ± 0.30 (500-700) 0.25 ± 0.015 (500-700) 0.035 ± 0.0021 (500-700)	0.3 1.47 1.4	A, B, C, D?, F, H?	-	iUCQY [100]
β-NaYF_4_: (18%)Yb^3+^,(2%)Er^3+^	Powder (In RI matched media	Several µm (*Varied)	T: 10 mm	*>0.1	980 (*Gauss)	17 ± 3 0.17 ± 0.03 0.025 ± 0.004	2.4 ± 0.14 (500-700) 0.062 ± 0.0037 (500-700) 0.0095 ± 0.00056 (500-700)	0.14 0.37 0.38	A, B, C, D?, F, H?	-	iUCQY [100]
β-NaYF_4_: (18%)Yb^3+^,(2%)Er^3+^	Powder (In air)	Several µm (*Varied)	T: 10 mm	*>0.1	980 (*Gauss)	17 ± 3 0.17 ± 0.03 0.025 ± 0.004	5.16 ± 0.3 (500-700) 0.78 ± 0.046 (500-700) 0.13 ± 0.0079 (500-700)	0.30 4.59 5.2	A, B, C, D?, F, H?	C	iUCQY [100]
NaBiF_4_: Tm^3+^	Powder	*<400 nm (*Varied)	-	*>0.1	980 (*Gauss)	400	2.49 (*400-900)	6.2 x10^−3^	A, B, C?, D?, F, H?, I, J	C?	eUCQY [141]
LiYF_4_: Er^3+^,Yb^3+^	Powder	L: ~2.0 µm (Octahedral)	-	*>0.1	980 (*Gauss)	-	2.1 (*350-750)	-	A, B, C?, D?, F?, H?, I, J	C?	eUCQY [142]
SrF_2_: (14.2%)Er^3+^	Powder	-(*Varied)	-	*>0.1	980 (*Gauss)	25 63	0.17 (*350-700) 0.28 (*350-700)	6.8 x10^−3^ 4.4 x10^−3^	A, B, C?, D?, F, H?, I, J	C?	eUCQY [143]
SrF_2_: (2%)Yb^3+^,(2%)Er^3+^	Powder	100-300 nm (Spherical and pseudo-cubic)	-	*>0.1	980 (*Gauss)	10	2.8 (*400-900)	0.28	A, B, C?, D?, F, H?, I, J	C?	eUCQY [88]
La_2_O_3_: (2%)Yb^3+^, (1%)Er^3+^	Powder	-(*varied)	-	*>0.1	980 (*Gauss)	7.6	3.8 (500-700)	0.5	A, B, C?, D?, H?, I, J	C?	iUCQY [93]
La_2_O_2_S: (1%)Yb^3+^, (1%)Ho^3+^	Powder	4.9 µm (Varied)	-	*>0.1	980 (*Gauss)	0.5	0.12 (*700-800)	0.24	A, B, C?, D?, H?, I, J	C?	eUCQY [144]
La_2_O_2_S: (9%)Yb^3+^,(1%)Er^3+^	Powder	-(*varied)	-	*>0.1	980 (*Gauss)	0.5 ± 0.01 3.8 ± 0.07 13 ± 0.26	0.4 ± 0.06 (350-900) 0.13 ± 0.02 (*750-900) 0.21 ± 0.03 (*600-700) 0.062 ± 0.009 (*500-600) 1.4 x10^−3^ ± 2 x10^−4^ (*350-450) 4.87 ± 0.7 (350-900) 1.03 ± 0.2 (*750-900) 3.13 ± 0.5 (*600-700) 0.69 ± 0.1 (*500-600) 0.02 ± 0.003 (*350-450) 5.83 ± 0.9 (350-900) 1.10 ± 0.2 (*750-900) 3.84 ± 0.6 (*600-700) 0.87 ± 0.10 (*500-600) 0.024 ± 0.04 (*350-450)	0.8 0.26 0.42 0.124 2.8 x10^−3^ 1.3 0.27 0.82 0.18 5.3 x10^−3^ 0.45 0.085 0.30 0.067 1.9 x10^−3^	A, B, C?, D?, F, H?, G	C?	eUCQY [136]
La_2_O_3_: (4%)Yb^3+^,(0.4%)Tm^3+^	Powder	2-5 µm (*Varied)	-	*>0.1	980 (*Gauss)	7.6	3.4 (750-850)	0.45	A, B, C?, D?, H?, I, J	C?	iUCQY [87]
La_1.76_Yb_0.18_Er_0.06_S_3_	Powder	-(*Varied)	L:300 mm	*>0.1	971 (*Gauss)	2.42	0.181 (*510-570) 0.028 (*640-720)	0.075 0.012	A, B, C?, D?, H?, I, J	C?	eUCQY [145]
La_2_O_3_: (18%)Yb^3+^,(2%)Er^3+^	Powder	-(*Varied)	Epoxy encapsulated 1×1 mm^2^ glass capillary	*>0.1	980 (*Ellip)	1.1 ± 0.2 CPD	0.2 (510−542), 0.04 ((820−870) 1.2 (Theoretical saturation)	0.18 0.036	A, B, C?, D?, H?, J	C?	eUCQY [67]
La_2_O_2_S: (1%)Yb^3+^	Powder	*~5±2 µm (Hexagonal)	-	*>0.1	980 (*Gauss)	22 ± 3	6.20 ±0.90 (350-900) 1.69 ± 0.25 (*750-880) 2.83 ± 0.40 (*600-750) 1.69 ± 0.30 (*500-600) 0.011 ± 0.002 (*350-450)	0.28 0.077 0.13 0.077 5 x10^−4^	A, B, C?, D?, F, H?, G	C?	eUCQY [146]
Y_2_O_2_S: (1%)Yb^3+^,(1%)Er^3+^	Powder	5±2 µm (Hexagonal)	-	*>0.1	980 (*Gauss)	19 ± 3	5.50 ± 0.80 (350-900) 2.44 ± 0.36 (*750-880) 1.52 ± 0.22 (*600-750) 1.53 ± 0.22 (*500-600) 0.019 ± 0.003 (*350-450).	0.29 0.13 0.08 0.081 1 x10^−3^	A, B, C?, D?, F, H?, G	C?	eUCQY [146]
Y_2_O_3_: (2%)Er^3+^	Powder	D: 0.7, L: 1-10 µm (Microtubes)	Round vial, D: 0.6 mm	*>0.1	980 (Gauss)	753	0.0056 (*500-700)	7.44 x10^−6^	A, B, C?, D?, F, H?, I, J	-	eUCQY [101]
ZrO_2_: (28%)Yb^3+^	Powder	150±60 µm (Varied)	-	*~0.25	976 (Gauss)	800	12.5 (380-900)	0.016	A, B, C?, D?, F, H?, I, J	C?	eUCQY [147]
ZrO_2_: (28%)Er^3+^	Powder	-(*Varied)	-	*>0.1	976 (Gauss)	800	10.1 (380-900)	0.013	A, B, C?, D?, F, G, H?	C?	eUCQY [147]
Gd_2_O_2_S: (3%)Yb^3+^	Powder	*~5±2 µm (Hexagonal)	-	*>0.1	980 (*Gauss)	9 ± *3	4.20 ± 0.60 (350-900) 1.88 ± 0.26 (*750-880) 1.53 ± 0.23 (*600-750) 0.75 ± 0.11 (*500-600) 0.024 ± 0.04 (*350-450)	0.47 0.21 0.17 0.083 2.7 x10^−3^	A, B, C?, D?, H?, I, J	C?	eUCQY [146]
Gd_2_O_2_S: Yb^3+^,Er^3+^	Powder	-(*Varied)	-	*>0.1	980 (*Gauss, 0.9 x1.3 mm)	1	1 (500-700 nm)	1	A, B, C?, D?, H?, I, J	C?	iUCQY [93]
YF_3_: (18%)Yb^3+^,(2%)Er^3+^	Powder	-(*Varied)	Epoxy encapsulated 1×1 mm^2^	*>0.1	980 (*Ellip)	1 ± 0.2 CPD	0.2 (510−542) 0.1 (820−870) 1.7 (Theoretical saturation)	0.2 0.1	A, B, C?, D?, H?, J	C?	eUCQY [67]
YCl_3_: (18%)Yb^3+^,(2%)Er^3+^	Powder	-(*Varied)	Epoxy encapsulated 1×1 mm^2^ glass capillaries	*>0.1	980 (*Ellip)	0.8 ± 0.1 CPD	0.2 (510−542) 0.01 (820−870) 1.2 (Theoretical saturation)	0.25 0.013-	A, B, C?, D?, H?, J	C?	eUCQY [67]
Ba_5_Gd_8_Yn_4_O_21_: (12%)Yb^3+^,(4%)Er^3+^	Powder	-(*Varied)	-	*>0.1	980 (*Gauss)	6.7	2.9 (500-700)	0.43	A, B, C?, D?, H?, I, J	C?	iUCQY [93]
Ba_4_Y_3_F_17_: (27%)Yb^3+^,(5%)Er^3+^	Powder	<1 µm (columnar and tabular grains)	-	*>0.1	973 (*Gauss)	*15	0.12 ±*0.03 (*400-420) 2.71 ±*0.68 (*500-580) 3.76 ±*0.94 (*620-690) 1.16 ±*0.29 (*800-900) 3.87 ±*0.97 (*500-580 + *800-900)	8 x10^−3^ 0.18 0.25 0.077 0.26	A, B, C?, D?, F, H?, I	C?	eUCQY [148]
BaY_2_ZnO_5_: (7%)Yb^3+^,(3%)Er^3+^	Powder	-(*Varied)	-	*>0.1	980 (*Gauss)	6.7	3.1 (500-700)	0.46	A, B, C?, D?, H?, I, J	C?	UCQY [93]
CaWO_4_: (0.25%)Ho^3+^,(2.5%)Yb^3^	Powder	-(*Varied)	-	*>0.1	974 (*Gauss)	47	3.3 (*350-775)	0.07	A, B, C?, D?, F, H?, I, J	C?	eUCQY [149]
50SiO_2_-20Al_2_O_3_-25CaF_2_-1Tb^3+^−5Yb^3+^	Co-doped glass	-	T: 2 mm	*>0.1	974 (*Gauss)	40–96	order of 10^−4^ (*450-700)	-	A, C?, D?, F, H?, I, J	C?	eUCQY [90]

### 4.2. UCQY measurements of nanoscale UC materials

In this sub-section, UCQY characterizations of nanoscale UC materials are subject to a similar review process, as seen in Table 3. It is important to note that many of the discussed influential effects are minimized when characterizing colloidally stable UCNPs, giving them higher comparability than their bulk counterparts. In fact, an encouraging report by Meijer et al. provided evidence of similar UCQYs obtained independently by three different labs when they characterized a common sample of colloidally stable LiYF_4_:Yb^3+^,Tm^3+^ UCNPs [78]. The UCQY were in good agreement between the labs. However, a small UCQY difference was obtained between labs that utilized a top-hat excitation beam profile and those that used a Gaussian beam profile, but this is understandable given the events discussed previously. Such analysis is promising for the feasibility of UCQY standards. However, this type of collaborative effort will have to occur more frequently for establishing them.

**Table 3.** Reviewing the reported values and measurement parameters for UCQY characterisations of nanoscale UC materials

**Sample**	**Type**	**Particle Size** **[shell]** **(Shape)**	**Dim**. **(Con.)**	**Abs.**	**Ex. λ** **[nm]** **(BP)**	**PD** **[W/cm^2^]**	**UCQY [%](Em. λ [nm])**	**N. UCQY** **[cm^2^/W]**	**Effects**	**Ad.**	**Cate-gory** **[Ref.]**
β-NaYF_4_: (17%)Yb^3+^,(3%)Er^3+^	Powder	~25 nm (Monodisperse, hexagonal)	H:: 30 mm, V: 1200 mm³	*>0.1	976 (TH)	20 (Stability < 0.1 %) 46 (Stability < 0.1 %)	0.32 (394-430) 0.039 (510-570) 0.015 (630-685) 0.14 (783-833) 0.11 (833-880) 0.0028 (370-890) 0.63 (394-430) 0.066 (510-570) 0.023 (630-685) 0.32 (783-833) 0.2 (833-880) 0.0079 (370-890)	0.016 2 x10^−3^ 7.5 x10^−4^ 7 x10^−3^ 5.5 x10^−3^ 1.4 x10^−4^ 0.014 1.4 x10^−3^ 5 x10^−4^ 7 x10^−3^ 4.3 x10^−3^ 1.7 x10^−4^	B, C?, D?, H, J	H, C?	eUCQY [15]
β-NaYF_4_: (17%)Yb^3+^,(3%)Er^3+^	CD in Toluene	~25 nm (Monodisperse, hexagonal)	H:: 30 mm, V: 1200 mm³	0.02	976 (TH)	20 (Stability < 0.1 %) 410 (Stability < 0.1 %)	3.7 x10^−4^ (394-430) 0.04 (510-570) 0.033 (630-685) 6.8 x10^−3^ (783-833) 0.015 (833-880) 0.098 (370-890) 0.037 (394-430) 0.43 (510-570) 0.75 (630-685) 0.04 (783-833) 0.14 (833-880) 1.44 (370-890)	1.85 x10^−5^ 2 x10^−3^ 1.65 x10^−3^ 3.4 x10^−4^ 7.5 x10^−4^ 4.9 x10^−3^ 9.02 x10^−5^ 1.1 x10^−3^ 1.8 x10^−3^ 9.8 x10^−5^ 3.4 x10^−4^ 3.5 x10^−3^	H, J	H	iUCQY (PS REF) [115]
β-NaYF_4_: (15%)Yb^3+^,(20%)Er^3+^	CD in Toluene	~28 nm (Hexagonal)	T: 1 cm (1 mg/mL)	~0.005	970 (Gauss)	181	0.11 (510-680)	6.08 x10^−4^	A, H?, I	-	iUCQY [91]
NaYF_4_: (15%)Yb^3+^,(2%)Er^3+^	CD in Toluene	~28 nm (Hexagonal)	1 cm cuvette (1 mg/mL)	~0.005	976 140 fs pulse width, 80 MHz (*Gauss)	187	0.49 (510-680)	2.62 x10^−3^	A, H?, I	-	iUCQY [91]
NaYF_4_: (18%)Yb^3+^,(2%)Er^3+^ @NaYF_4_	Powder	23 [45] nm (Hexagonal)	-	*>0.1	976 (TH)	20-40	~9 (370-890)	~0.3	B, C?, D?, F, H?, I, J	C?	iUCQY (PS REF) [115]
β-NaY_0.8_F_4_:Yb_0.18_Er_0.02_	CD in Cyclohexane	8−22 nm (*Hexagonal)	Cylindrical quartz cuvette	-	977.5 (TH-like)	63 ± 7	0.045 (*500-700) ~0.025 (*500-600) ~0.02 (*600-700)	7.14 x10^−4^ 3.97 x10^−4^ 3.17 x10^−4^	A, G, H?	-	iUCQY (PS REF) [80]
β-aY_0.72_Gd_0.04_Lu_0.04_F_4_:Yb_0.18_Er_0.0_	CD in Cyclohexane	8−22 nm (*Hexagonal)	Cylindrical sample holder T: 3mm	-	977.5 (TH-like)	63 ± 7	0.074 (*500-700) ~0.04 (*500-600) ~0.032 (*600-700)	1.17 x10^−3^ 6.35 x10^−4^ 5.08 x10^−4^	A, G, H?	-	iUCQY (PS REF) [80]
β-NaYF_4_: (20%)Yb^3+^,(2%)Ho^3+^@β-NaYF_4_	CD in CHCl_3_	D: ~23 [38] nm (*Rectangular)	-	-	976	100	0.591 (*460-700)	5.91 x10^−3^	A, H?, I, J	-	iUCQY (PS REF) [113]
OA-coated β-NaYF_4_: (20%)Yb^3+^,(2%)Er^3+^	CD in D_2_O (DPSE shell)	22.7 ± 0.7 nm (Hexagonal)	-	0.02	976 (*Gauss)	~10^4^	~1 (*400-900)	1 x10^−4^	A, E, F, H?, I	E, F	eUCQY [129]
β-NaYF_4_: (33%)Yb^3+^,(5%)Er^3+^@β-NaLuF_4_	CD in Chloroform	~23.8 [~36.8] nm (Nearly spherical)	-	-	980 (Gauss + TH)	63 ± 7	1.47 ± 0.16 (*500-600) 2.58 ± 0.29 (*600-700) 4.04 ± 0.45 (*500-700)	0.023 0.041 0.064	A, H	H	iUCQY (PS REF) [17]
NaYF_4_: (18)%Yb^3+^,(2%)Er^3+^	CD in Water	31.9 ± 9.0 nm (Quasi-spherical + hexagonal)	(16.5 mg/mL)	-	980 (*Gauss)	753.08	~5 x10^−3^ (400-700)	6.64 x10^−6^	A, E, F, H?, I, J	-	iUCQY [99]
β-NaYF_4_: (2%)Er^3+^,(20%)Yb^3+^	CD in Hexane	100 nm (Hexagonal)	-	-	980 (*TH, D: 1 mm)	150	0.3 ± 0.1 (*500-600)	2 x10^−3^	B, H?, I	-	iUCQY (PS REF) [84]
β-NaYF_4_: (2%)Er^3+^,(20%)Yb^3+^	CD in Hexane	30 nm (Hexagonal)	-	-	980 (*TH, D: 1 mm)	150	0.1 ± 0.05 (*500-600)	6.67 x10^−4^	H?, I	-	iUCQY (PS REF) [84]
β-NaYF_4_: (2%)Er^3+^,(20%)Yb^3+^	CD in Hexane	(8-10) nm (Hexagonal)	-	-	980 (*TH, D: 1 mm)	150	0.005 ± 0.005 (*500-600)	3.33 x10^−5^	H?, I	-	iUCQY (PS REF) [84]
β-NaYF_4_: (2%)Er^3+^,(20%)Yb^3+^@NaYF_4_	CD in Hexane	30 nm (Hexagonal)	-	-	980 (*TH, D: 1 mm)	150	0.3 ± 0.1 (*500-600)	2 x10^−3^	H?, I	-	iUCQY (PS REF) [84]
β-NaYF_4_: (20%)Yb^3+^,(2%)Er^3+^	CD in Hexane	~5.4 nm (Hexagonal)	-	-	980 (*Gauss)	10^3^	0.0022 ± 0.0001 (490-700)	2.2 x10^−6^	A, H?, I	-	eUCQY [188]
β-NaYF_4_: (20%)Yb^3+^,(2%)Er^3+^@NaYF_4_	CD in Hexane	~5.4 [~9] nm (Hexagonal)	-	-	980 (*Gauss)	10^3^	0.18 ± 0.01 (490-700)	1.8 x10^−4^	A, H?, I	-	eUCQY [188]
β-Na(Y_0.68_/Lu_0.12_)F_4_:Yb_0.18_Er_0.02_	CD in IPA (ATTO 542-coated)	~25 nm (Hexagonal)	-	-	977.5 (*Gauss)	50 (Stability: 0.185%)	*~0.029 (*500-700)	5.8 x10^−4^	A, B?, F?, G, H?	-	iUCQY (PS REF) [28]
β-NaGdF_4_: (20%)Yb^3+^,(2%)Er^3+^@NaYF_4_	CD in Cyclohexane	3.7 ± 0.5 [*~13.7] nm (Hexagonal)	-	-	980 (*Gauss)	420	1.7 (*400-900)	4.05 x10^−3^	H?, I	-	iUCQY (*PS REF) [116]
Β-NaGdF_4_: (~22%)Yb^3+^,(~2.2%)Er^3+^ @NaYF_4_ (Homogeneous doping)	CD in Cyclohexane	~2 [10] nm (Hexagonal)	-	-	980 (*Gauss)	50	0.89 ± 0.05 (*400-750)	0.018	A, H?, I	-	eUCQY [189]
Β-NaGdF_4_: (~22%)Yb^3+^,(~2.2%)Er^3+^ @NaYF_4_ (Heterogeneous doping)	CD in Cyclohexane	~2 [10] nm (Hexagonal)	-	-	980 (*Gauss)	50	0.47 ± 0.05 (*400-750)	9.4 x10^−3^	A, H?, I	-	eUCQY [189]
β-NaGdF_4_: Yb^3+^,Er^3+^	CD in Cyclohexane	~5 nm (Hexagonal)	-	-	980 (*Gauss)	*100	0.016 ± 0.08 (*400-750)	1.6 x10^−4^	A, H?, I	-	eUCQY [178]
β-NaGdF_4_: Yb^3+^,Er^3+^@NaYF_4_	CD in Cyclohexane	~5 [~17] nm (Hexagonal)	-	-	980 (*Gauss)	*100	0.51 ± 0.08 (*400-750)	5.1 x10^−3^	A, H?, I	-	eUCQY [178]
NaYF_4_: (20%)Yb^3+^,(1.2)%Tm^3+^	CD in Toluene	~35 nm (Quasi-spherical + hexagonal plate-like)	-	--	980nm (TH)	140	8.4 ± 0.6 (334-890) *~8 (720-890) *~0.015 (675-720) *~0.048 (462-500) *~0.02 (433-462) *~1 x10^−3^ (334-353)	0.06 0.057 1.07 x10^−4^ 3.43 x10^−4^ 1.43 x10^−4^ 7.14 x10^−6^	H?, I	-	iUCQY (PS REF) [114]
β-NaLuF_4_: (24%)Gd^3+^,(20%)Yb^3+^,(1%)Tm^3+^	Powder	7.8 nm (Hexagonal)	-	*>0.1	980 (*Gauss)	17.5	0.47 ± 0.06 (*750-850)	0.027	A, B, C?, D?, F, H?, I	C?	iUCQY [190]
PAA modified NaBiF_4_:Tm^3+^	Powder	*<200 nm (*Varied)	-	*>0.1	980 (*Gauss)	400	3.7 (*400-900)	9.3 x10^−3^	A, B, C?, D?, F, H?, I, J	C?	eUCQY [179]
LiYF_4_:(25%)Yb^3+^,(0.5%)Tm^3+^ (Leiden lab)	CD in Toluene	LD: 87 ± 9 nm, SD: 50 ± 4 nm (Tetragonal)	(~40 mg/mL)	-	~969 (Near-Gauss)	0.07 ±0.0011 1 ± 0.015 5 ± 0.075 50 ± 0.75	(9 ± 3) x10^−3^ (430-860) (9 ± 3) x10^−3^ (720–890) (19 ± 6) x10^−3^ (430-860) (6 ± 2) x10^−7^ (433–462) (210 ± 6) x10^−7^ (462–500) (11 ± 4) x10^−6^ (610–675) (19 ± 6) x10^−3^ (720–890) (26 ± 8) x10^−3^ (430-860) (7 ± 2) x10^−6^ (433–462) (7 ± 2) x10^−5^ (462–500) (3.3 ± 1) x10^−5^ (610–675) (26 ± 8) x10^−3^ (720–890) 0.035 ± 0.011 (430-860) (23 ± 7) x10^−5^ (433–462) (28 ± 9) x10^−5^ (462–500) (14 ± 5) x10^−5^ (610–675) 0.035 ± 0.011 (720–890)	0.13 0.13 0.019 6 x10^−7^ 2.1 x10^−5^ 1.1 x10^−5^ 0.019 0.0052 1.4 x10^−6^ 1.4 x10^−5^ 6.6 x10^−6^ 5.2 x10^−3^ 7 x10^−4^ 4.6 x10^−6^ 5.6 x10^−6^ 2.8 x10^−6^ 7 x10^−4^	A, H?	-	iUCQY [78]
LiYF_4_:(25%)Yb^3+^,(0.5%)Tm^3+^ (Berlin lab)	CD in Toluene	LD: 87 ± 9 nm, SD: 50 ± 4 nm (Tetragonal)	(~40 mg/mL)	-	~969 (Near-Gauss)	5.5 ± 0.0055 48 ±0.048 212 ± 0.212 395 ± 0.395	(189 ± 5) x10^−4^ (430-860) 3 x10^−6^ (433–462) (448 ± 2) x10^−7^ (462–500) (254 ± 3) x10^−7^ (610–675) 0.0188 (720–890) (260 ± 6) x10^−4^ (430-860) (908 ± 5) x10^−7^ (433–462) (165 ± 1) x10^−6^ (462–500) (848 ± 5) x10^−7^ (610–675) 0.0255 (720–890) (34 ± 2) x10^−3^ (430-860) 7.1 x10^−4^ (433–462) (515 ± 3) x10^−6^ (462–500) 2.63 x10^−4^ (610–675) 0.0310 (720–890) (371 ± 7) x10^−4^ (430-860) 1.37 x10^−3^ (433–462) (878 ± 2) x10^−6^ (462–500) (424 ± 1) x10^−6^ (610–675) 0.0325 (720–890)	3.44 x10^−3^ 1.09 x10^−6^ 8.15 x10^−6^ 4.62 x10^−6^ 3.42 x10^−3^ 5.4 x10^−4^ 1.9 x10^−6^ 3.4 x10^−6^ 1.8 x10^−6^ 5.3 x10^−4^ 1.6 x10^−4^ 3.4 x10^−6^ 2.4 x10^−6^ 1.2 x10^−6^ 1.5 x10^−4^ 9.4 x10^−5^ 1.8 x10^−6^ 2.2 x10^−6^ 1.1 x10^−6^ 8.2 x10^−5^	H?	-	iUCQY [78]
LiYF_4_: Er^3+^,Yb^3+^	Powder	L: 16 nm (Octahedral)	-	*>0.1	976 (*Gauss)	-	0.04 (*350-750)	-	A, B, C?, D?, F, H?, I, J	C?	eUCQY [180]
LiYbF_4_: (0.5%)Tm^3+^@LiYF_4_	CD in Hexane	11.5 ± 1.3 [26.4 ± 1.6] nm (Bipyramidal)	-	-	960 (*Gauss)	90	0.6 ±*0.06 (332-830) (49 ±*4.9) x10^−3^ (461-502) (21 ±*2.1) x10^−3^ (609-692)	6.67 x10^−3^ 5.44 x10^−4^ 2.33 x10^−4^	A, H?, I	-	iUCQY (PS REF) [6]
LiYbF_4_: (5%)Er^3+^@LiYF_4_	CD in Hexane	10.4 ± 0.7 [22.6 ± 1.4] nm (Bipyramidal)	-	-	960 (*Gauss)	90	0.78 ±*0.078 (374-713) (10 ±*1) x10^−4^ (374-392) (14 ±*1.4) x10^−3^ (396-428) 0.17 ±*0.017 (511-581) 0.59 ±*0.059 (625-713)	8.67 x10^−3^ 1.11 x10^−5^ 1.56 x10^−4^ 1.89 x10^−3^ 6.56 x10^−3^	A, H?, I	-	iUCQY (PS REF) [6]
LiYbF_4_: (5%)Ho^3+^@LiYF_4_	CD in Hexane	12 ± 1.2 [25.4 ± 1.2] nm (Bipyramidal)	-	-	960 (*Gauss)	90	(74 ±*7.4) x10^−3^ (468-763) (90 ±*9) x10^−5^ (468-503) (39 ±*3.9) x10^−3^ (513-569 + 736-763) (35 ±*3.5) x10^−3^ (614-693)	8.22 x10^−4^ 1 x10^−5^ 4.33 x10^−4^ 3.89 x10^−4^	A, H?, I	-	iUCQY (PS REF) [6]
LiYbF_4_: (2%)Er^3+^@LiYF_4_	Powder	~30 nm (Rhombohedral)	-	*>0.1	980 (*Gauss)	70	3.36 ± 0.06 (410-690)	0.048	A, B, C?, D?, F, H?, I, G	C?	iUCQY [86]
LiYbF_4_: (2%)Ho^3+^@LiYF_4_	Powder	~30 nm (Rhombohedral)	-	*>0.1	980 (*Gauss)	70	0.69 ± 0.01 (470-630)	9.86 x10^−3^	A, B, C?, D?, F, G, H?, I	C?	iUCQY [86]
LiYbF_4_: (1%)Tm^3+^@LiYF_4_	Powder	~30 nm (Rhombohedral)	-	*>0.1	980	70 (*Gauss)	0.81 ± 0.06 (330-840)	0.012	A, B, C?, D?, F, G, H?, I	C?	iUCQY [86]
La_0.75_Yb_0.20_Tm_0.05_F_3_ (150 mg) and La_0.20_Yb_0.75_Er_0.05_F_3_ (1 mg)	Silica sol-gel thin film	~8 nm (*Near spherical)	(150 nm)	*>0.1	980 (*Gauss)	-	0.10 ± 0.01 (*500-600)	-	A, B, C?, D?, E?, F? H?, I	C?	iUCQY [191]
SrF_2_: (20%)Yb^3+^,(2%)Er^3+^	CD in Water	~40 nm (*Cubic)	(2.5 mg/mL)	-	980 (Near-Gauss)	390 ± 30	(2.8 ± 0.1) x10^−3^ (*500-700)	7.18 x10^−6^	A, E, F, H?	-	eUCQY [131]
ZnMoO_4_: (0.3%)Er^3+^,(7.5%)Yb^3+^,(10%)K^+^	Powder	*~100 nm (Plates and rectangles)	-	~40%	980 (*Gauss)	0.01	0.31±0.02 (400-700)	31	A, B, C?, D?, F, G, H?, I	C?	eUCQY [192]
Mo_4_O_15_:Yb_1.82_Er_0.18_	NC thin film	1-20 nm (*Varied)	-	*>0.1	975 (*Gauss)	~0.5	~1.3 (500–575)	2.6	A, B, C?,D?, H?, I, J	C?	iUCQY [193]
NaGdF_4_: (18%)Yb^3+^,(2%)Er^3+^@NaYF_4_	CD in Toluene	5.5 [16] nm (Hexagonal)	-	*>0.1	980 (*Gauss)	100	~0.22 (500-700) Extrapolated data	2.2 x10^−3^	A, H, I, J	H	*eUCQY [135]
SrLuF: (30.6%)Yb^3+^,(2.9%)Er^3+^@SrLuF	CD in Toluene	4.9 (L: ~11] nm (Face-centred cubic)	-	*>0.1	980 (*Gauss)	100	~0.66 (500-700) Extrapolated data	6.6 x10^−3^	A, H, I, J	H	*eUCQY [135]
SrLuF: (30.6%)Yb^3+^,(2.9%)Er^3+^@SrLuF	CD in Toluene	4.9 (~16] nm (Face-centred cubic)	-	*>0.1	980 (*Gauss)	100	~1 (500-700) Extrapolated data	0.01	A, H, I, J	H	*eUCQY [135]

### 4.3. UCQY measurements of UC materials excited by Short-Wave Infrared (1300-2500 nm) radiation

It has been reported that the largest UCQY values achievable in various Er^3+^-doped samples arises from the ^4^I1_1/2_→^4^I_15/2_ transition, where excitation is in the shortwave infrared (1300–2500 nm) and emission occurs ~980 nm. This is due to the high absorption peak of these materials in this region and the inclusion of the prominent UC emission peak ~980 nm. Such a characterization is important to review for advancing applications such as UC-PV devices [136]. Specifically, 1493 nm and 1523 nm have been targeted in Er^3+^-doped samples using monochromatic excitation to achieve high quantum efficiencies due to the material’s high resonance and absorption at these wavelengths [104,137–139]. However, as highlighted by MacDougall et al. energy level broadening of each interacting level makes UC achievable through broadband excitation using a bandwidth of 100 nm around 1523 nm [140]. Through experimental investigation of β-NaYF_4_:(10%)Er^3+^ phosphor embedded in perfluorocyclobutyl (PFCB), these authors found that the sample’s UCQY has a dependency on the excitation bandwidth. Using an excitation of 227 ± 10 W/cm^2^ centred around 1523 nm, the material exhibited an UCQY (~940-1040 nm) of 8.7 ± 0.3% using an excitation beam FWHM of 12 ± 0.5 nm, and 16.2 ± 0.5% when the FWHM was 61 ± 0.5 nm. It was explained that greater resonance and larger peaks occur at wavelengths on the shorter side of 1523 nm. By increasing the excitation bandwidth on this side, a larger portion of excitation can be utilised and therefore, there is greater population of the ^4^I_13/2_ level. This level is used for GSA and is necessary for the UC transition of interest. Additionally, in comparison to monochromatic excitation, which achieves the highest UCQYs by targeting the wavelength of maximum resonance and exploiting ETU as the most dominant mechanism, broadband sources excite multiple resonant peaks. This can lead to an improved photoluminescence performance through higher contributions of the ESA mechanisms. Since the excitation wavelength has already been highlighted as a crucial parameter to report, this effect was not included in Table 1. However, researchers should be aware of the large UCQY differences that can arise due to subtle differences in the bandwidth when using broadband excitation. UCQYs obtained under these excitation conditions are presented in Table 4. Through excitation ~1500 nm, multiple sources have reported significant UCQYs (900–1100 nm) of >10% using monocrystalline BaY_2_F_8_:Er^3+^ materials, which are recognised as one of the most efficient in the field [83,104,111]. Furthermore, under 1511 nm excitation (power density = 4070 ± 210 W/m^2^), Gd_2_O_2_S:(10%)Er^3+^ and Gd_2_O_2_S:(5%)Er^3+^ revealed substantially high iUCQYs (500–1050 nm) of 15.1 ± 1.4% and 15.3 ± 1.1%, respectively [13,141]. A iUCQY value of 16.2 ± 0.5% using 1523 nm excitation (power density = 227 ± 10 W/cm^2^), was recorded by MacDougall et al. when analysing β-NaYF_4_:(10%)Er^3+^ phosphor [140]. However, this group was the first to highlight the effects of reemission, where they showed that such a high UCQY is misleading as the perceived sample absorption is being influenced due to downshifting emission photons and therefore a correction is required [83]. As discussed earlier, these corrections significantly reduce the UCQY magnitude.

**Table 4.** Reviewing the reported values and measurement parameters for absolute UCQY characterisations of UC materials excited in the SWIR (1300–2500 nm) region

**Sample**	**Type**	**Particle Size** **[w/shell]** **(Shape)**	**Dim**. **(Con.)**	**Abs.**	**Ex. λ** **[nm]** **(BP)**	**PD** **[W/cm^2^]**	**UCQY [%](Em. λ [nm])**	**N. UCQY** **[cm^2^/W]**	**Effects**	**Ad.**	**Cate-gory** **[Ref.]**
BaY_2_F_8_: (0.5%)Er^3+^	Mono- crystalline	-	D: 4 ± 0.229 mm T: 2 ± 0.229 mm	*>0.1	1493 (*Gauss)	(62 ± 4.5) x10^−3^	8 x10^−3^ ± 1 x10^−3^ (940-1040)	0.13	A, C?, D?, F, H?	C?	eUCQY [159]
β-NaYF_4_: (10%)Er^3+^	Powder embedded in PFCB	-(Hexagonal)	T: 1 mm D: 10 mm	*>0.1	~1458-1580 (Broad)	227 ± 10	16.2 ± 0.5 (*940-1040)	0.0714	A, B, D?, E, F, H?	-	iUCQY [160]
β-NaYF_4_: (10%)Er^3+^	Powder embedded in PFCB	-(Hexagonal)	T: 1 mm D: 10 mm	*>0.1	~1507-1531 (Broad)	227 ± 10	8.7 ± 0.3 (*940-1040)	0.0383	A, B, D?, E, F, H?	-	iUCQY [160]
BaY_2_F_8_: (0.5%)Er^3+^	Mono- crystalline	-	D: 4 ± 0.229 mm T: 2 ± 0.229 mm	*>0.1	1493 (*Gauss)	(62 ± 4.5) x10^−3^	(582 ± 6) x10^−3^ (940-1040)	9.39	A, C?, D?, F, H?	C?	eUCQY [159]
β-NaYF_4_: (10%)Er^3+^	Powder embedded in PFCB	-(Hexagonal)	T: 1 mm D: 12.5 mm (55.6 w/w% in the matrix)	*>0.1	~1443-1603 (Broad)	197 ± 24	*~9 (*940-1040)	~0.0457	A, B, D?, E, F, H, J	H	[83]
β-NaYF_4_: (20%)Er^3+^	Powder	-(Hexagonal)	-	*>0.1	1523 (*Gauss) 1500 – 1550 (Broad) 1450 - 1600 (Broad)	(89.3 ± 5.3) x10^−3^ (65.11 ± 4.69) x10^−3^ 0.15743 ± 0.01134	2.73 ± 0.28 (*940-1040) 1.03 ± 0.17 (*940-1040) 0.99 ± 0.18 (*940-1040)	30.57 15.82	A, B, C?, D?, H? A, B, D?, H? A, B, D?, H?	C?	eUCQY [75]
β-NaYF_4_: (20%)Er^3+^	Powder embedded in PFCB	-(Hexagonal)	T: 1 mm D: 12.5 mm (55.6 w/w% in the matrix)	*>0.1	~1443-1603 (Broad)	197 ± 24	10.7 ± 1.2 (*940-1040)	0.054	A, B, D?, E, F, H	H	iUCQY [194]
BaY_2_F_8_: (20%)Er^3+^	Mono- crystalline	-	0.49 ± 0.01 mm	*>0.1	1493 (*Gauss)	7.0 ± 0.7	14.6 ± 1.5 (*900-1100)	0.054	A, C?, D?, H?	C?	iUCQY [111]
β-NaYF_4_: (22.5%)Er^3+^	Powder	-(Hexagonal)	-	*>0.1	1523 (Gauss)	0.40 ± 0.02	13.8 ±1.0 (*500-1050)	34.5	A, B, C?, D?, F, H?,	C?	eUCQY [162]
β-NaYF_4_: (25%)Er^3+^	Powder	~100 μm (Hexagonal)	-	*>0.1	1510 / 1523 (*Gauss)	(70 ± 5.6) x10^−3^	8.9 ± 0.7 (440-1080)	127.14	A, B, C?, D?, F, H?, J	C?	iUCQY [163]
β-NaYF_4_: (25%)Er^3+^	Powder embedded in PFCB	-(Hexagonal)	T: 1 mm D: 12.5 mm (84.9 w/w% in the matrix)	*>0.1	1523 (10.5 ± 10^−4^ cm^2^, *Gauss)	(97 ± 4.3) x10^−3^	8.4 ± 0.8 (*940-1040)	86.6	A, B, C?, D?, E, F, H?	C?	iUCQY [102]
β-NaYF_4_: (25%)Er^3+^	Powder embedded in PFCB	-(Hexagonal)	T: 1 mm D: 12.5 mm (55.6 w/w% in the matrix)	*>0.1	~1443-1603 (Broad)	126 ± 15 65 ± 8 44 ± 5 26 ± 3 11 ± 1	8.7 ± 1 (*940-1040) 8.8 ± 0.9 (*940-1040) 6 ± 0.7 (*940-1040) 5 ± 0.6 (*940-1040) 2 ± 0.2 (*940-1040)	0.069 0.135 0.136 0.192 0.182	A, B, D?, E, F, H	H	iUCQY [161]
β-NaYF_4_: (25%)Er^3+^	Powder	-(Hexagonal)	-	*>0.1	1508 (*Gauss) 1500–1550 (Broad) 1450–1600 (Broad)	(94.1 ± 5.7) x10^−3^ (69.5 ± 5) x10^−3^ 0.1677 ± 0.0121	3.53 ± 0.36 (*940-1040) 1.98 ± 0.32 (*940-1040) 1.87 ± 0.31 (*940-1040)	37.51 28.49 11.15	A, B, C?, D?, H? A, B, D?, H? A, B, D?, H?	C?	eUCQY [75]
β-NaYF_4_: (25%)Er^3+^	Powder	20-200 µm (Hexagonal)	3mm thick compressed layer in metal cylinder	*>0.1	1523 (*Gauss)	0.402 ± 0.021	12.0 ± 1 (*500-1050)	29.85	A, B, C?, D?, F, H?	C?	iUCQY [13]
β-NaYF_4_: (25%)Er^3+^	Powder	20-200 µm (Hexagonal)	3mm thick compressed layer in metal cylinder	*>0.1	1450-1600 (Broad)	0.1675 ± 0.012	1.86 ± 0.31 (*500-1050)	11.11	A, B, D?, F, H?	-	eUCQY [13]
β-NaYF_4_: (40%)Er^3+^	Powder embedded in PFCB	-(Hexagonal)	T: 1 mm D: 12.5 mm (55.6 w/w% in the matrix)	*>0.1	~1443-1603 (Broad)	197 ± 24	*~6 (*940-1040)	~0.0305	A, B, D?, E, F, H, J	H	[83]
β-NaYF_4_: (28%)Er^3+^ @β-NaLuF_4_	CD in CHCL_3_	~19.2 [~29] nm (Hexagonal)	-~5.8 × 10^14^ UCNCs/mL	Absorptance:~15.0 ± 3.3	1523 (*Gauss)	0.43 ± 0.03	0.716 ± 0.178 (*500-1100)	1.65	A,H?	-	iUCQY [68]
β-NaYF_4_: (28%)Er^3+^ @β-NaLuF_4_	NCs embedded in PMMA	~19.2 [~29] nm (Hexagonal)	-2.7 × 10^14^ UCNCs/mL	Absorptance:~4.3 ± 0.5	1523 (*Gauss)	0.43 ± 0.03	1.96 ± 0.21 (*500-1100)	4.67	A, B, C?, D?, E, F, H?	C?	iUCQY [68]
BaY_2_F_8_: (30%)Er^3+^	Mono- crystalline	-	D: 10 ± 0.229 mm T: 2 ± 0.229 mm	*>0.1	1493 (*Gauss)	(62 ± 4.5) x10^−3^	3.62 ± 0.01 (940-1040)	58.39	A, C?, D?, F, H?	C?	eUCQY [159]
BaY_2_F_8_: (30%)Er^3+^	Mono- crystalline	-	1.75 x 4.26 x 4.41 mm^3^	*>0.1	1520 (*Gauss)	0.474± 0.025	10.1 ± 1.6 (*500-1100)	21.31	A, C?, D?, F, H?	C?	iUCQY [104]
BaY_2_F_8_: (30%)Er^3+^	Mono- crystalline	-	1.75 ± 0.01 mm	*>0.1	1493 (*Gauss)	7.0 ± 0.7	12.1 ± 1.2 (*900-1100)	1.73	A, C?, D?, H?	C?	eUCQY [111]
Gd_2_O_2_S: (5%)Er^3+^	Powder	-(Hexagonal)	-	*>0.1	1511 (*Gauss)	0.47 ±0.03	15.3 ±1.1 (*500-1050)	32.55	A, B, C?, D?, F, H?	C?	iUCQY [162]
Gd_2_O_2_S: (5%)Er^3+^	Powder	2-10 μm (Hexagonal)	-	*>0.1	1510 / 1523 (*Gauss)	(70 ± 5.6) x10^−3^	12 ± 1 (440-1080)	171.43	A, B, C?, D?, F, H?, J	C?	eUCQY (Low doped reference) [195]
Gd_2_O_2_S: (5%)Er^3+^	Powder	2-10 μm (Hexagonal)	3mm thick compressed layer	*>0.1	1511 (*Gauss)	0.474± 0.025	15.1 ± 1.4 (*500-1050)	37.10	A, B, C?, D?, F, H?	C?	iUCQY [13]
Gd_2_O_2_S: (5%)Er^3+^	Powder	2-10 μm (Hexagonal)	3mm thick compressed layer	*>0.1	1450-1600 (Broad)	0.1675 ± 0.012	1.09 ± 0.18 (*500-1050)	6.51	A, B, D?, F, H?	-	eUCQY [13]
SrF_2_: (5%)Er^3+^,(1%)Yb^3+^	Powder	-	-	*>0.1	~1500 (*Gauss)	-	0.20 (*400-690)	-	A, B, C?, D?, F?, H?, I, J	C?	eUCQY [196]
SrF_2_: (8.8%)Er^3+^	Powder	-	-	*>0.1	~1500 (*Gauss)	510	0.19 (*360-720)	3.73 x10^−4^	A, B, C?, D?, F?, H?, I, J	C?	eUCQY [197]

### 4.4. UCQY measurements of UC materials excited around the 800 nm NIR region

Interest has grown around UC materials excited ~800 nm because of their high potential in the biomedical field due to the optical transparency of organic matter in this region. Consequently, an UCQY review on materials characterised in this way is presented in Table 5. The magnitude of these UCQYs is significantly less compared to those acquired under SWIR excitation, for the reasons previously described. Furthermore, although limited data is available, the reliability of these characterisations are affected by many of the same effects.

**Table 5.** Reviewing the reported values and measurement parameters for absolute UCQY characterisations of UC materials excited ~800 nm

**Sample**	**Type**	**Particle Size** **[w/shell]** **(Shape)**	**Dim**. **(Con.)**	**Abs.**	**Ex. λ** **[nm]** **(BP)**	**PD** **[W/cm^2^]**	**UCQY [%](Em. λ [nm])**	**N. UCQY** **[cm^2^/W]**	**Effects**	**Ad.**	**Cate-gory** **[Ref.]**
NaYF_4_:Yb^3+^,Er^3+^ @NaYF_4_:Yb^3+^ @NaNdF_4_:Yb^3+^ @NaYF_4_ @NaGdF_4_	CD in Cyclohexane	19.6 ± 1.6 [47.2 ± 5.1] nm (Hexagonal)	(1 mg/mL)	-	800 (*Gauss)	17	0.75 ± 0.08 (400-700)	0.044	A, H?, I	-	eUCQY [198]
β-NaYF_4_:(20%)Yb^3+^,(2%)Er^3+^ @NaYF_4_:(10%)Yb^3+^ @NaNdF_4_:(10%)Yb^3+^	CD in Cyclohexane	~7 [~16] nm (Hexagonal)	(20 mg/mL)	-	800 (*Gauss)	20	0.11 ± 0.05 (*500-600)	5.5 x10^−3^	A, H?, I	-	iUCQY [89]
NaYF_4_:(20%)Yb^3+^,(2%)Er^3+^@NaYF_4_:(10%)Yb^3+^ @NaNdF_4_:(10%)Yb^3+^ @NaYF_4_:(10%)Yb^3+^	CD in Chloroform	[*<50] nm (Hexagonal)	-	-	808 (*Gauss)	31	0.18 (*500-600)	5.81 x10^−3^	A, H?, I, J	-	eUCQY [199]
NaYF_4_:Yb^3+^,Er^3+^ @NaYF_4_:Nd^3+^,Yb-ADA	CD in *Cyclohexane (with antenna IRDye)	~24nm [~29.4] nm (*Hexagonal)	-	-	808 (*Gauss)	1.5	2.48 (*535-570)	1.65	A, H?, I, J	-	iUCQY [200]
NaYF_4_:Gd^3+^ @NaYF_4_:Yb^3+^,Er^3+^ @NaYF_4_:Nd^3+^,Yb^3+^-ADA	CD in *Cyclohexane (with antenna IRDye)	~20.6 [~25.6] nm (*Hexagonal)	-	-	808 (*Gauss)	1.5	3.33 (*535-570)	2.22	A, H?, I, J	-	iUCQY [200]
IR-806-β-NaYF_4_:(20%)Yb^3+^,(2%)Er^3+^ @β-NaYF_4_:(10%)Yb^3+^	CD in DMF (IR-806 sensitized)	20.3 ± 1.6 [35.9 ±1.4] nm (Hexagonal)	Cylindrical cuvette (0.01 μmol/L)	-	800 (*Gauss)	2	~5 (450−720)	2.5	A, D, H?, I, J	-	iUCQY (PS REF) [177]

### 4.5. UCQY measurements of UC materials enhanced through plasmonic structures, photonic structures, and dye sensitizers

In this sub-section, the UCQY literature regarding UC nanomaterials enhanced by plasmonic structures, photonic structures, or organic sensitizers, is reviewed. In relation to the former, it should be noted that these structures do not always enhance the UCQY and the exact influence they have on the radiative and nonradiative decay rates of UC materials is still being debated [142]. As first pointed out by Wu et al. in 2014, most UCQY characterisations in the field of plasmonically enhanced UC materials lack characterisation of the associated power density or only report the value alongside a single measurement of power [143]. The UCQY prior to the plasmonic enhancement effects is also under reported, which also needs to be addressed since less efficient UC materials are generally prone to greater enhancements compared to materials with efficiencies that are initially high in relation [143]. Our analysis has revealed that the UCQYs in this sub-field are currently predominantly based on rate equation methods [26,144–151]. This is understandable due to influence that plasmonic structures can have on the radiative and non-radiative decay rates however, it creates difficulties for direct comparisons with absolute UCQY values. Moving forward, it would be beneficial to the field to obtain data on the absolute UCQY magnitudes achievable when exploiting these materials. These future characterisations should clearly define the materials compositions used, their geometries, as well as the distance between them as this is related to the Forster resonance energy transfer (FRET) mechanism [144,152]. Furthermore, the analysis should also be conducted on a control sample that does not contain the plasmonic material so that an intrinsic UCQY can be stated.

Photonic structures have also been developed for the purpose of enhancing the photoluminescence efficiency of UC nanomaterials [153–155]. Predominantly, UCQY studies in this sub-field are also conducted using rate equation methods [27,156–158]. As a result, similar conclusions are drawn.

Organic dye molecules have been exploited as NIR sensitizers to enhance the efficiency of UC materials [159]. For example, the tailored NIR dye transfers energy through a multi-step nonradiative process to nearby sensitizer ions embedded in a nanostructure shell [160]. Then, a subsequent energy transfer occurs between these ions and RE^3+^ ions that are doped into the core of the structure. Interestingly, this creates a route for exciting these RE^3+^ ions through a broader range of NIR excitation. However, the combination of two different photoluminescent species creates new challenges for reporting the UCQY. Although limited examples are available, there are two instances where these problems have been addressed [28,117]. Wisser et al. reported that the ePLQY of Na(Y/Gd/Lu)F_4_:(18%)Yb^3+^,(2%)Er^3+^ UCNPs was increased by a factor of 10 by decorating their surfaces with these types of commercial dye (ATTO 542) sensitizers [28]. To do so, the authors separated the Er^3+^ green UC emission, Er^3+^ red UC emission, and the dye’s emission into separate PLQY contributions. Although not addressed, uncertainties arise due to the overlapping of the UC emission and the dye’s emission as well as the self-absorption effect that likely limits the dyes emission magnitude. Although they should be mentioned, obtaining reliable corrections for these effects would indeed be challenging.

### 4.6. UCQY measurements of organic UC materials

Despite focusing on RE^3+^-doped inorganic UC materials, the authors acknowledge the potential of organic UC molecules, which rely on a mechanism known as triplet-triplet UC (TTA-UC) [161]. These materials also require thorough UCQY studies and the information in this review is relevant to them also. From our examination of the literature, the UCQY measurements of TTA-UC materials are commonly undertaken using relative means, despite the reliability issues associated with these methods [66,161–172]. In 2019 however, Yanai et al. established the first absolute UCQY determinations of TTA-UC materials to address this issue [173]. These authors also highlight a couple interesting pieces of information regarding the measurements. Firstly, they acknowledge the influence of UC emission self-absorption and detail a correction method to amend for it. Secondly, they note a problematic issue that has arisen in this sub-field relating to the multiplication of UCQYs by a factor of two to normalize the maximum UC efficiency of a two-photon process to 100%. They condone this practice due to its misleading nature when comparing between studies.

## 5. Conclusion

### 5.1. Influential effects and the path to obtaining reliable UCQY standards

Effects primarily related to the non-linear power dependency of the UC mechanism create added challenges that traditional PLQY methodologies do not adequately prepare researchers for. Furthermore, the reporting of experimental values alongside the UCQY is varied between groups, making it hard to determine the influence of certain affects and direct comparisons between UCQY values misleading. To address this, a variety of effects that should be considered by researchers when determine the UCQY are described and summarized in this work. Furthermore, a template is provided for researchers to follow for reporting all experimental parameters that have importance for comparability. In association with this, the UCQY standard is defined, and a path is established for achieving its characterization. These values are reserved for UCQYs with the highest comparability, after all associated effects have been addressed. Ideally, these standards will be reported over a broad range of excitation power densities until UCQY saturation occurs to be as informative as possible. The material properties that should be reported alongside the UCQY standard include its exact composition, particle size, particle concentration, and the samples absorbance. The dimensions of the sample inside the integrating sphere are also relevant as well as the dispersion or encapsulating media used. Furthermore, various experimental parameters should be stated, such as the excitation power density, the excitation beam profile, and the volume of sample that it probes, excitation FP position, the excitation wavelength and its integration range, the emission wavelength and its integration range, the equipment used, the integrating sphere type and size, the optical cuvette type, the reference sample used and its composition, as well as the sample temperature. Finally, the UCQY and associated power density should be accompanied by statistical errors and a complete list of effects that are influencing the result should be stated. It is understood however that due to differences in sample preparation, measurement equipment, calibration procedures, and correction files for the instrument response at various wavelengths, there will unlikely be UCQY standards that are accurate to a high number of significant figures. Although, it is very encouraging for the field that different labs were able to obtain similar UCQYs for a common UCNP sample [78].

The main challenges associated with UCQY measurements involve the characterization of bulk UC materials. This is due to the heightened significance of scattering effects, beam profile distortion effects, inner-filter effects, particle concentration effects, thermal effects, and sample geometry, which are commonly minimized when studying their UCNP counterparts. Achieving reliable corrections for these effects, which occur simultaneously and are related to each other in some cases, is a very challenging pursuit. Instead, their UCQYs should be reported in a manner that adequately reflects the influence of these effects so that they are understood when attempts are made to repeat similar characterizations.

### 5.2. Current UCQY literature and future characterisations

Having summarized the most significant effects, previously reported UCQY values were categorized depending on how they addressed them as well as the experimental parameters that were reported alongside them. Some values appeared to have higher comparability than others due to the low significance of these effects. An iUCQY (370–890 nm) of 0.098% was reported for β-NaYF_4_:(17%)Yb^3+^,(3%)Er^3+^ UCNPs using a power density of 20 W/cm^2^ (λ_Ex_ = 976 nm) [15]. In this instance, the small size of the UCNPs minimized effects such as scattering and the primary inner-filter effect. Additionally, the samples low absorbance was beneficial for minimizing UC emission self-absorption, indirect excitation, and particle concentration effects. Furthermore, the excitation possessed a top-hat beam profile, therefore power density variations were minimized throughout the sample. Additionally, beam profile distortion effects were not considered due to the sample’s low absorption. These review tables presented can also be used to compare which UC materials have shown the highest efficiencies. Although defining the most efficient UC material is challenging, the highest UCQY reported appears to originate from the characterization of Gd_2_O_2_S:(5%)Er^3+^ [141]. Here, an iUCQY(500–1050 nm) of 15.3 ± 1.1% was observed at an excitation power density of 4070 ± 210 W/m^2^ (λ_Ex_ = 1511 nm), although various effects are influencing the result.

In terms of UC materials enhanced by plasmonic and photonic structures, absolute UCQY is absent from the field as the efficiency characterizations are primarily acquired using rate equation methods. Obtaining absolute UCQY of these materials would be beneficial to the field and allow for more comparable results with other UC materials. When obtaining these results, researchers should report the absolute UCQY before and after plasmonic effects to clearly define the UC efficiency enhancement achieved. Additionally, the UCQY study should involve a broad range of excitation power densities to be as informative as possible on the materials potential. Similar conclusions are drawn regarding UC materials enhanced by dye-sensitizer molecules. Finally, it was highlighted that absolute UCQY studies have begun to arise in the field of TTA-UC materials [173]. This is important as it moves the field away from relative methods, which struggle due to reliability issues. Absolute UCQY data is required on a broader range of TTA-UC molecules to make more comprehensive comparisons. These future characterizations can use the information in this work to report these values with the highest possible comparability.

Without addressing the issues relating to UCQY measurements, the UC field will struggle to advance at its current pace. Questions will remain regarding the optimal performances achievable in UC materials. Additionally, difficulties will continue to arise when distinguishing the efficiency benefits of new UC material compositions compared to materials currently available. As such, future work involves addressing these characterization issues and then determining a range of UCQY standards for various UC materials that can be reliably contrasted to.

## Supplementary Material

Supplemental Material
