# Supplementary material for: The upconversion quantum yield (UCQY): a review to standardize the measurement methodology, improve comparability, and define efficiency standards
Source: Sci Technol Adv Mater. 2021 Dec 17;22(1):810–48. doi: 10.1080/14686996.2021.1967698 (PMC8725918; doi:10.1080/14686996.2021.1967698)
Supplement: Supplemental Material [file TSTA_A_1967698_SM1567.docx]

Supporting information

The upconversion quantum yield (UCQY): a review to standardize the measurement methodology, improve comparability, and define efficiency standards

Callum M. S. Jones ^1^, Anna Gakamsky ^2^, and Jose Marques-Hueso ^1,^*

^1^Institute of Sensors, Signals and Systems, Heriot-Watt University, Edinburgh, United Kingdom

^2^Edinburgh Instruments Ltd., Kirkton Campus, Livingston, United Kingdom

E-mail: * [j.marques@hw.ac.uk](mailto:j.marques@hw.ac.uk)

# Contents

Reviewing UCQYs determined through non-absolute methods

# Reviewing UCQYs determined through non-absolute methods

| **Category**  **[Ref.]** | (Power efficiency method)  [1] | (Internal standard method)  [2] | (Relative method)  [3] | (Internal standard method)  [2] | (Relative method)  [4] | (Relative method)  [4] | (Internal standard method)  [2] | (Relative method)  [5] |
| --- | --- | --- | --- | --- | --- | --- | --- | --- |
| **Ad.** | - | - | - | - | - | - | - | - |
| **Effects** | K | K | K | K | K | K | K | K |
| **N. UCQY**  **[cm^2^/W]** | 3.62  2.48  1.14 | 3.27 x10^-4^  7.51 x10^-3^  0.010 | 0.56 | 1.73 x10^-3^  0.037  0.041 | 1.18 x10^-3^ | 9.23 x10^-3^ | 1.62 x10^-3^  1.73 x10^-3^  0.13 | 0.045 |
| **UCQY [%] (Em. λ [nm])** | 3.62 (*400–600)  2.48 (*600-700)  1.14 (*500-600) | 0.0235 ± 0.01 (*400-440)  0.54 ± 0.02 (*500-600)  0.72 ± 0.02 (*600-700) | ~4-5 (*300-700) | 0.126 ± 0.004 (*400-440)  2.68 ± 0.08 (*500-600)  3 ± 0.09 (*600-700) | 0.45 (*700-900) | 1.2 (*700-900) | 0.127 ± 0.004 (*400-460)  0.135 ± 0.004 (*460-500)  10.44 ± 0.01 (*750-900) | 3.5 (*700-900) |
| **PD**  **[W/cm^2^]** | 1 | 503 @936 nm, which is equivalent to 71.9 @976 nm | ~8 | 509 @936 nm, which is equivalent to 72.7 @976 nm | 3.8 x10^-4^  BPD | 1.3 x10^-4^  BPD | 547 @936 nm, which is equivalent to 78.2 @976 nm | 78 |
| **Ex. λ**  **[nm]**  **(BP)** | 974  (*Gauss) | Equivalent to 976  (Gauss) | 800  (*Gauss) | Equivalent to 976  (Gauss) | 975  (*Gauss) | 975  (*Gauss) | Equivalent to 976  (Gauss) | 975  (*Gauss) |
| **Abs.** | - | ≤0.004 (@ 936 nm) | - | ≤0.004 (@ 936 nm) | - | - | ≤0.004 (@ 936 nm) | - |
| **Dim.**  **(Con.)** | - | (≤12 mg mL−1) | - | (≤12 mg mL−1) | - | - | (≤12 mg mL−1) | - |
| **Particle Size**  **[w/shell]**  **(Shape)** | 50–90 nm  (Hexagonal) | W: 55.9 ± 3.1  L: 54.0 ± 1.6 nm  (Hexagonal) | ~26  [~33] nm  (Hexagonal) | W: 55.9 ± 3.1  (65.8 ± 3.1)  L: 54.0 ± 1.6  (64.2 ± 2.9)  nm (Hexagonal) | ~33 nm  (Hexagonal) | ~33  [~ 43] nm  (Hexagonal) | W: 71.8 ± 2.5  (89.6 ± 2.5 )  L: 68.4 ± 1.3  (79.6 ± 2.4)  nm (Hexagonal) | ~36  [~6] nm  (Hexagonal) |
| **Type** | Powder | CD in  Toluene | CD in  DMF (indocya-nine green sensitized) | CD in Toluene | CD in  *Hexane | CD in  *Hexane | CD in Toluene | CD in  *Hexane |
| **Sample** | NaY_0.885_Y_0.1_Er_0.015_F_4_ | β-NaYF_4_:  (18%)Yb^3+^,(2%)Er^3+^ | NaYF_4_:  (20%)Yb^3+^,(2%)Er^3+^  @NaYF_4_:(20%)Yb^3+^ | β-NaYF_4_:  (18%)Yb^3+^,(2%)Er^3+^  @NaYF_4_ | NaYF_4_:  Yb^3+^,Tm^3+^ | NaYF_4_:  Yb^3+^,Tm^3+^@NaYF_4_ | β-NaYF_4_:  (25%)Yb^3+^,(0.5%)Tm^3+^  @NaYF_4_ | β-NaYF_4_:  Yb^3+^,Tm^3+^@NaYF_4_ |

Table. S1. Reviewing the reported values and measurement parameters for non-absolute UCQY characterisations of UC materials.

| **Category**  **[Ref.]** | (Relative method)  [6] | (Internal standard method)  [2] | (Relative method)  [7] | (Relative method)  [8] | (Relative method)  [9] | (Relative method)  [10] | (Power efficiency method)  [11] | (Relative method)  [12] | (Relative method)  [12] | (Relative method)  [12] | (Relative method)  [12] |
| --- | --- | --- | --- | --- | --- | --- | --- | --- | --- | --- | --- |
| **Ad.** | - | - | - | - | - | - | - | - | - | - | - |
| **Effects** | K | K | K | K | K | K | K | K | K | K | K |
| **N. UCQY**  **[cm^2^/W]** | 0.029 | 4.49 x10^-4^  4.19 x10^-4^  0.049 | 2 | 1 x10^-4^ | 0.48 | 8.67 x10^-4^  4.67 x10^-4^  3.33 x10^-4^  6.33 x10^-3^  8 x10^-3^ | 2.5 x10^-3^ | 1.65 x10^-6^  1.85 x10^-5^ | 0.01  5 x10^-4^ | 8.5 x10^-7^  5 x10^-6^ | 9 x10^-3^ |
| **UCQY [%] (Em. λ [nm])** | 0.4 (*500-900) | 0.0343 ± 0.001 (*400-460)  0.0320 ± 0.001 (*460-500)  3.71 ± 0.1 (*750-900) | 0.6 ± 0.1 (700-900) | 0.1 (*~355-400) | 4.8 (*300-670) | 0.13 ± 0.02 (*500-600)  0.07 ± 0.02 (*600-700)  0.05 ± 0.01 (*750-900)  0.95 ± 0.05 (*900-1100)  1.2 ± 0.1 (*500-1100) | 0.1 (*500-750) | 3.3 x10^-5^ (*500-600)  3.7 x10^-4^ (*600-700) | 0.2 (*500-600)  0.01 (*600-700) | 1.7 x10^-5^ (*425-525)  1 x10^-4^ (*500-600) | 0.18 (*425-525) |
| **PD**  **[W/cm^2^]** | 14 | 534 @936 nm, which is equivalent to 76.4 @976 nm | 0.3 | 10^3 | 10 | 150 | 40 | 20 | 20 | 20 | 20 |
| **Ex. λ**  **[nm]**  **(BP)** | 975  (Gauss) | Equivalent to 976)  (Gauss) | 975  (Gauss) | 975  (Gauss, 0.374 cm^2^) | 800  (*Gauss) | 1490  (*Gauss) | 980  (*Gauss) | 976  (*Gauss) | 976  (*Gauss) | 976  (*Gauss) | 976  (*Gauss) |
| **Abs.** | - | ≤0.004 (@ 936 nm) | 0.09 | - | - | - | - | - | - | - | - |
| **Dim.**  **(Con.)** | - | (≤12 mg mL−1) | - | (10 mg/mL) | - | - | - | - | - | - | - |
| **Particle Size**  **[w/shell]**  **(Shape)** | ~32  [~42] nm  (Hexagonal) | W: 71.8 ± 2.5  L: 68.4 ± 1.3  nm (Hexagonal) | ~20  [~27] nm  (*Cubic) | ~22  [~27] nm  (Cubic) | ~44  [∼54] nm  (Hexagonal) | ~85 nm  (Spindle shape) | ~35 nm  (Monoclinic phase) | ~48 nm  (*Varied) | ~57 nm  (*Varied) | ~48 nm  (*Varied) | ~56 nm  (*Varied) |
| **Type** | CD in  Cyclohexane | CD in  Toluene | CD in  Hexane | CD in  Hexane | CD in DMF (dye-sensitized) | CD in  Chloroform | Powder | Powder | Powder | Powder | Powder |
| **Sample** | NaYF_4_:  Yb^3+^,Tm^3+^@NaYF_4_ | β-NaYF_4_:  (25%)Yb^3+^,(0.5%)Tm^3+^ | α-NaYbF_4_:  (0.5%)Tm^3+^@CaF_2_ | α-NaYbF_4_:  Tm^3+^@CaF_2_ | NaYbF_4_:  (0.5%)Tm^3+^@NaYF_4_:(30%)Nd^3+^ | LiYF_4_:  (10%)Er^3+^ | YNbO_4_:  (10%)Yb^3+^,(2%)Er^3+^ | Er_0.25_Yb_2.75_Al_5_O_12_ | Er_0.25_Yb _2.25_Mo _0.5_Al_5_O_12_ | Tm_0.25_Yb_2.75_Al_5_O_12_,YbAG:Tm | Tm_0.25_Yb_2.25_Mo_0.5_Al_5_O_12_ |

# References

1. Yasyrkina D, Kuznetsov S, Ryabova A, et al. Dependence of quantum yield of up-conversion luminescence on the composition of fluorite-type solid solution nay 1-x-yyb XEr YF 4. Наносистемы: физика, химия, математика. 2013;4(5).

2. May PS, Baride A, Hossan MY, et al. Measuring the internal quantum yield of upconversion luminescence for ytterbium-sensitized upconversion phosphors using the ytterbium (iii) emission as an internal standard. Nanoscale. 2018;10(36):17212-17226.

3. Chen G, Shao W, Valiev RR, et al. Efficient Broadband Upconversion of Near‐Infrared Light in Dye‐Sensitized Core/Shell Nanocrystals. Advanced Optical Materials. 2016;4(11):1760-1766.

4. Liu H, Xu CT, Lindgren D, et al. Balancing power density based quantum yield characterization of upconverting nanoparticles for arbitrary excitation intensities. Nanoscale. 2013;5(11):4770-4775.

5. Xu CT, Svenmarker P, Liu H, et al. High-resolution fluorescence diffuse optical tomography developed with nonlinear upconverting nanoparticles. ACS nano. 2012;6(6):4788-4795.

6. Mousavi M, Thomasson B, Li M, et al. Beam-profile-compensated quantum yield measurements of upconverting nanoparticles. Physical Chemistry Chemical Physics. 2017;19(33):22016-22022.

7. Chen G, Shen J, Ohulchanskyy TY, et al. (α-NaYbF4: Tm3+)/CaF2 core/shell nanoparticles with efficient near-infrared to near-infrared upconversion for high-contrast deep tissue bioimaging. ACS nano. 2012;6(9):8280-8287.

8. Shen J, Chen G, Ohulchanskyy TY, et al. Tunable near infrared to ultraviolet upconversion luminescence enhancement in (α‐NaYF4: Yb, Tm)/CaF2 Core/Shell nanoparticles for In situ real‐time recorded biocompatible photoactivation. small. 2013;9(19):3213-3217.

9. Chen G, Damasco J, Qiu H, et al. Energy-cascaded upconversion in an organic dye-sensitized core/shell fluoride nanocrystal. Nano letters. 2015;15(11):7400-7407.

10. Chen G, Ohulchanskyy TY, Kachynski A, et al. Intense visible and near-infrared upconversion photoluminescence in colloidal LiYF4: Er3+ nanocrystals under excitation at 1490 nm. ACS nano. 2011;5(6):4981-4986.

11. Tian Y, Tian Y, Huang P, et al. Effect of Yb3+ concentration on upconversion luminescence and temperature sensing behavior in Yb3+/Er3+ co-doped YNbO4 nanoparticles prepared via molten salt route. Chemical Engineering Journal. 2016;297:26-34.

12. Dong B, Cao B, He Y, et al. Temperature sensing and in vivo imaging by molybdenum sensitized visible upconversion luminescence of rare‐earth oxides. Advanced Materials. 2012;24(15):1987-1993.
